# Identification of microRNAs Targeting the Transporter Associated with Antigen Processing TAP1 in Melanoma

**DOI:** 10.3390/jcm9092690

**Published:** 2020-08-20

**Authors:** Maria-Filothei Lazaridou, Chiara Massa, Diana Handke, Anja Mueller, Michael Friedrich, Karthikeyan Subbarayan, Sandy Tretbar, Reinhard Dummer, Peter Koelblinger, Barbara Seliger

**Affiliations:** 1 Institute of Medical Immunology, Martin Luther University Halle-Wittenberg, Magdeburger Str. 2, 06112 Halle, Germany; marifili.lazaridou@uk-halle.de (M.-F.L.); chiara.massa@uk-halle.de (C.M.); diana.handke@uk-halle.de (D.H.); anjamueller@uk-halle.de (A.M.); michael.friedrich@uk-halle.de (M.F.); karthik.subbarayan@uk-halle.de (K.S.); sandy.tretbar@uk-halle.de (S.T.); 2 Institute of Dermatology, University Hospital Zürich, 8091 Zürich, Switzerland; Reinhard.Dummer@usz.ch; 3 Department of Dermatology and Allergology, University Hospital Salzburg, 5020 Salzburg, Austria; p.koelblinger@salk.at

**Keywords:** immune escape, microRNA, melanoma, transporter associated with antigen processing

## Abstract

The underlying molecular mechanisms of the aberrant expression of components of the HLA class I antigen processing and presentation machinery (APM) in tumors leading to evasion from T cell-mediated immune surveillance could be due to posttranscriptional regulation mediated by microRNAs (miRs). So far, some miRs controlling the expression of different APM components have been identified. Using in silico analysis and an miR enrichment protocol in combination with small RNA sequencing, miR-26b-5p and miR-21-3p were postulated to target the 3′ untranslated region (UTR) of the peptide transporter TAP1, which was confirmed by high free binding energy and dual luciferase reporter assays. Overexpression of miR-26b-5p and miR-21-3p in melanoma cells downregulated the TAP1 protein and reduced expression of HLA class I cell surface antigens, which could be reverted by miR inhibitors. Moreover, miR-26b-5p overexpression induced a decreased T cell recognition. Furthermore, an inverse expression of miR-26b-5p and miR-21-3p with TAP1 was found in primary melanoma lesions, which was linked with the frequency of CD8^+^ T cell infiltration. Thus, miR-26-5p and miR-21-3p are involved in the HLA class I-mediated immune escape and might be used as biomarkers or therapeutic targets for HLA class I^low^ melanoma cells.

## 1. Introduction

Human solid tumors including melanoma develop different strategies to escape T cell-mediated immune surveillance such as loss or downregulation of human leukocyte antigens (HLA) class I molecules. This is frequently due to defects in the expression of various components of the antigen processing and presentation machinery (APM), which can be associated with disease progression and reduced patient survival [1,2,3,4]. Alterations in the HLA class I pathway may be a result of the selective pressure of the immune system and could occur in patients treated with immunotherapies [5,6]. During the last few decades, the underlying molecular mechanisms of HLA class I and APM component deficiencies have been characterized demonstrating a high diversity, ranging from deregulation to rather rare structural alterations [3,7,8,9]. The deregulation of HLA class I APM components in tumors could occur at the transcriptional, epigenetic, posttranscriptional and/or posttranslational level and depend on the tumor type analyzed [2,10,11,12]. Recently, the posttranscriptional regulation of HLA class I APM components has come into focus, which could be mediated by RNA binding proteins (RBP) or small non-coding microRNAs (miRs) [13,14,15,16].

MiRs with a length of approximately 20–23 nucleotides belong to the family of small non-coding RNAs [17] and play an important role in the posttranscriptional control of gene expression by binding to the 5′ UTR, 3′ UTR or coding sequence of the targeted mRNAs [18] either preventing translation or inducing degradation [17]. A broad spectrum of miRs have been identified to be abnormally expressed in hematologic malignancies and solid tumors including melanoma [19]. Dependent on their targets, miRs could influence tumor formation, metastasis, disease progression as well as the composition of the immune cell infiltration of the tumor microenvironment (TME) [20]. They function as oncogenes or tumor suppressor genes, but could also participate in immune escape or altered immune responses by targeting or affecting the expression of different immune modulatory molecules in tumor and immune cells [14].

The implementation of different strategies, such as in silico prediction, miR arrays, small RNA sequencing, RNA affinity approaches and luciferase (luc) reporter gene assays, resulted in the identification and functional characterization of a small number of miRs targeting the 3′UTR of the HLA class I heavy chain (HC), HLA-G, TAP1 or TAP2 [21,22,23,24,25,26,27,28]. Due to this limited information, the identification of novel miRs involved in HLA class I mediated immune escape mechanisms is urgently needed and might lead to a better understanding of tumor development and progression as well as response or resistance to immunotherapies such as checkpoint inhibitors or adoptive cell therapy [29,30,31,32,33,34]. To identify novel and specific miRs, the miRNA trapping by RNA in vitro affinity purification (miTRAP) assay has been shown to provide a rapid, reliable and easy-to-handle protocol for the enrichment of regulatory miRs for RNAs of choice in the cellular context of interest [35,36]. Apart from the 3′UTR, the target sequences can be the 5′UTR or the coding sequence (CDS) [35]. MiRs targeting immune modulatory molecules might serve as prognostic and/or predictive tumor markers or be used as therapeutic tools alone or in combination with immunotherapies [14,37].

Malignant melanoma represents the most common skin cancer with an incidence that has rapidly increased over the past few decades [38,39]. The interaction of melanoma cells with cells of the TME influences the biology of melanoma cells, such as proliferation, differentiation and progression [40]. Furthermore, melanoma cells are often highly immunogenic and have the capacity to induce an adaptive immune response [41]. This might be due to their high mutational burden leading to the expression of tumor-associated antigens (TAA) [42,43]. However, melanoma cells escape T cell recognition by different mechanisms, like inefficient antigen processing and presentation, modulation of immune stimulatory or immune suppressive molecules and alterations in the cellular composition of the TME [29,44,45,46]. This knowledge has led to the approval of therapeutic approaches aiming to overcome immune evasion, i.e., anti-CTLA-4 and anti-PD1 antibodies [39]. Another possibility might be to target immune modulatory miRs that have been demonstrated to be involved in innate and adaptive immune responses [47].

Therefore, in this study, miTRAP combined with small RNA sequencing was employed to identify novel miRs targeting TAP1 [35,36,48]. Two selected miRs were functionally analyzed in melanoma cell lines. In addition, their clinical relevance regarding survival outcome in melanoma patients and association with tumor immune cell infiltration was determined.

## 2. Experimental Section

### 2.1. Cell Lines and Cell Culture Conditions

The human embryonic kidney cell line HEK293T and the human TAP-negative T2 cell lines (ATCC^®^ CRL-1992™) were obtained from the American Tissue Culture Collection (ATCC), the human melanoma cell lines FM3 (ESTDAB-007), FM81 (ESTDAB-026) and MZ-Mel2 (CVCL-1435) [49] from the European Searchable Tumour Cell Line and Data Bank (ESTDAB project; http://www.ebi.ac.uk/ipd/estdab) [50,51] and BUF1379 from Soldano Ferrone (Department of Surgery, Massachusetts General Hospital, Harvard Medical School, Boston, MA, USA). The HEK293T cells were cultured in Dulbecco’s Modified Eagles Medium (DMEM, Invitrogen, Carlsbad, CA, USA), while all the other cell lines were maintained in Roswell Park Memorial Institute 1640 medium (RPMI 1640, Invitrogen) supplemented with 10% (v/v) fetal calf serum (FCS) (PAN, Aidenbach, Germany), 2 mM L-glutamine (Lonza, Basel, Switzerland) and 1% penicillin/streptomycin (v/v, Sigma-Aldrich, Saint Louis, MO, USA) at 37 °C in 5% (v/v) CO_2_ humidified air. T2 cells negative for TAP1, TAP2, LMP2, LMP7 and MHC class II antigens served as a control for peptide pulsing [52]. T cells specific for the HLA-A2-restricted Melan A/Mart-1 epitope were kindly provided by Pedro Romero (Ludwig Institute for Cancer Research, Lausanne, Switzerland) and cultured in RPM 1640 supplemented with 8% human serum.

### 2.2. Human Melanoma Tissues

Tissue samples from cutaneous malignant melanoma used in this study (*n* = 20) were collected between 2008 and 2016 in the Department of Dermatology, University Hospital of Zurich, Zurich, Switzerland [53]. The study was performed according to the declaration of Helsinki and approved by the ethical committees of the University Hospital in Zurich (KEK-ZH-No. 647 and 800) as well as of the University Hospital in Salzburg (E-No. 2142). The clinical data from the melanoma patients as well as the PD-L1 expression and immune cell infiltration of the tumor lesions have recently been published [53].

### 2.3. Plasmids and Cloning

The recombinant vectors for luciferase reporter assays, oligonucleotide sequences used for PCR reactions and cloning strategies have recently been described [35,36,54] and are listed in Supplementary Table S1. The recombinant plasmid DNA pcDNA™ 3.1(+) (Invitrogen) with the two MS2 loops was kindly provided by Prof. Dr. Stefan Hüttelmaier (Institute of Molecular Medicine, Martin Luther University Halle-Wittenberg, Halle (Salle, Germany). All amplified inserts were sequenced for their identity prior to subcloning into the respective vectors.

### 2.4. MiRNA Trapping by RNA In Vitro Affinity Purification (miTRAP)

MiTRAP is a suitable method for the identification of miRs that specifically target immune relevant molecules. Detailed protocols for the different steps of this method have been recently published [35,36,48,55]. Briefly, the TAP1 3′ UTR (NM_000593.5) was cloned upstream of the coding sequence for two MS2 stem loop structures, in vitro transcribed with RiboMAX large scale RNA production system (Promega, Mannheim, Germany) and used for the enrichment of TAP1-specific miRs from cell lysates of MZ-Mel2 (CVCL_1435) [49]. Then, amylose resin beads (NEB) were washed and incubated with the fusion protein maltose-binding protein (MBP) fused to the MS2-binding protein, blocked with yeast tRNA (Invitrogen) and bovine serum albumin (BSA; Invitrogen) and incubated with the bait RNAs (TAP1 3′ UTR or the sequence encoding only the two MS2 loops (MS2) as a negative control) and the cell lysate. With the exception of cell lysis, all steps were performed at room temperature under constant agitation. For miR analysis, RNA complexes were eluted twice with 15 mM maltose and miR were purified from maltose solution by phenol–chloroform extraction. Untreated cell lysate was used for RNA extraction and applied as an input control. The miR enrichment in the eluates was further validated by RT-qPCR. For protein analysis, protein complexes were eluted once with Laemmli buffer and together with 1%, 0.5%, and 0.2% dilution series of the cell lysate, prepared as input controls, were subjected to Western blot analysis using 10% SDS-PAA gels.

### 2.5. Isolation of Plasmid DNA, Cellular RNA, miRNA and qPCR Analysis

Plasmid DNA was isolated either with NucleoSpin^®^ Plasmid or the NucleoBond^®^ Xtra Midi kits (Macherey-Nagel, Schkeuditz, Germany) for small or medium scale plasmid preparation, respectively. The NucleoSpin^®^ Gel and PCR Clean-up kit (Macherey-Nagel) was employed for the DNA purification of the PCR products and the digested plasmids with the respective restriction enzymes as recently described [56].

Total cellular RNA from cell cultures was isolated using the NucleoSpin RNA kit (Macherey-Nagel, Schkeuditz, Germany) according to the manufacturer’s instructions. Total cellular RNA and miR were extracted from cell cultures using the TRIzol reagent (Invitrogen) according to the manufacturer’s instructions. For RNA isolation from paraffin-embedded tissue sections, total RNA was extracted using the NucleoSpin Tissue kit (Macherey-Nagel). The isolated RNA was treated with DNaseI (New England Biolabs (NEB), Ipswich, MA, USA) for 30 min at 37 °C, inactivated with 50 mM EDTA for 10 min at 75 °C and then used as template for cDNA synthesis.

RT-qPCR was performed as previously described [51,57]. Briefly, RNA was reverse transcribed into cDNA using RevertAid^TM^ H Minus First Strand cDNA synthesis kit (Thermo Scientific, Waltham, MA, USA) together with oligo dT primers (Thermo Scientific) for mRNA and miR specific stem loop primers for the miR [57,58,59]. For qPCR reaction, the 2× SYBR Green qPCR Master Mix (Absource, Munich, Germany) was employed with target-specific primers (Supplementary Table S1). The reverse transcription reactions were carried out in a 96-well labcycler (Sensoquest, Göttingen, Germany) and the qPCR reactions in a BIO-RAD 96-well iCycler (BIO-RAD Laboratories, Inc., Hercules, CA, USA). For qPCR, the relative changes of RNA abundance were determined by the ΔCt method using the housekeeping genes glyceraldehyde-3-phosphate dehydrogenase (GAPDH) or β-actin (ACTB) for normalization, whereas the relative miR expression levels were normalized to the corresponding expression levels of the small non-coding RNA RNU6A. The reactions were performed at least in triplicates for each biological replicate.

### 2.6. Protein Extraction and Western Blot Analysis

For Western blot analysis, 50 μg protein/lane was separated in 10% SDS-PAGE gels, transferred onto nitrocellulose membranes (Schleicher & Schuell, Munich, Germany) and stained with Ponceau S performed as previously described [60]. Immunodetection was performed using the following specific primary antibodies (Ab): anti-TAP1 (ab13516, Abcam, Cambridge, UK), anti-TAP2, kindly provided by Soldano Ferrone, anti-AGO2 (ab156870, Abcam) and anti-MBP Abs (ab9084, Abcam). Staining with anti-GAPDH (#2118, Cell Signaling Technology, Danvers, MA, USA) or ACTB Ab (ab8227, Abcam) served as a loading control. The membranes were then stained with suitable horseradish peroxidase (HRP) conjugated secondary Abs (DAKO, Hamburg, Germany or Cell Signaling Technology, Danvers, MA, USA), before the signal was visualized with the Pierce Western Blot Signal Enhancer substrate (Thermo Scientific) and recorded with a LAS3000 camera system (Fuji LAS3000, Fuji GmbH, Düsseldorf, Germany) using the Image Reader LAS3000 software. The immunostaining signals were subsequently analyzed using the ImageJ software (NIH, Bethesda, Rockville, MD, USA). Relative protein expression levels are provided as arbitrary units by setting the peak values of the corresponding GAPDH signals to 1.

### 2.7. Luciferase Reporter Assay

The TAP1 3′ UTR was cloned in the pmiR-Glo Dual-Luciferase miRNA target expression vector (Promega, Madison, Washington, DC, USA) with the restriction enzymes NheI and SalI (Thermo Scientific) as recently described [56]. For the deletion of the binding side of miR-26b-5p or miR-21-3p in the TAP1 3′ UTR, specific primers were designed according to the NEBaseChanger software (https://nebasechanger.neb.com/, NEB) (Supplementary Table S1). The Q5^®^ Site-Directed Mutagenesis kit (NEB) was employed according to manufacturer’s instructions. On day zero, 1 × 10^4^ HEK293T cells/well were seeded into 96-well plates. After 12–16 h, the cells were co-transfected with 30 nM mimics (Sigma-Aldrich) and 5 ng recombinant TAP1 3′ UTR pmiR-Glo vector using Lipofectamin 2000 (Invitrogen). The cells were washed with phosphate-buffered saline (PBS) 48 h post-transfection and lysed in lysis buffer (Promega). The firefly and renilla luciferase (luc) activities were determined using the DualGlo reagent (Promega) with the GloMax 96-Microplate luminometer (Promega) kindly provided by Prof. Guido Posern (Institute of Biophysical Chemistry, Martin Luther University Halle-Wittenberg, Halle (Saale, Germany) with the Dual-Luciferase^®^ Reporter Assay System (Promega) according to manufacturer’s instructions. Firefly luc (FFL) activities were normalized to Renilla luc (RL) activities yielding relative light units (RLU). The empty pmiR-Glo vector only containing the multiple cloning site served as a negative control. RLU ratios were normalized to control populations. All experiments were performed at least three times in triplicates.

### 2.8. Transfection of miR

To determine the impact of miR on the expression of HLA class I APM components, 3 × 10^5^ cells were seeded in 6-well plates and transiently transfected after 12–16 h with mimics (30 nM) or inhibitors (100 nM) (miR-26b-5p, miR-21-3p or the negative control (NC), Sigma-Aldrich) using 9 μL Lipofectamine RNAiMAX (Invitrogen) according to the manufacturer’s instructions. The cells were harvested 48 h post-transfection for subsequent qPCR, Western blot, flow cytometric and functional analyses.

### 2.9. Flow Cytometry

The monoclonal antibodies (mAb) employed for flow cytometry were the PE-Cyanine7-labelled anti-HLA-ABC (BioLegend, San Diego, CA, USA), the APC-labelled anti-HLA-BC (BioLegend), the unconjugated anti-HLA-A2 (kindly provided by Soldano Ferrone) and the PE-labelled-secondary goat anti-mouse Ab (Jackson ImmunoResearch, Cambridgeshire, UK) at concentrations recommended by the manufacturers. Briefly, 1–5 × 10^5^ cells were incubated with the appropriate amounts of respective Ab at 4 °C in darkness for 30 min. The stained cells were measured on a BD FACS LSRFortessa (Becton Dickinson (BD), New Jersey, NJ, USA) and subsequently analyzed with the FACS Diva analysis software (BD). The data are expressed as mean specific fluorescence intensities (MFI).

### 2.10. CD107a Degranulation Assay

Tumor cell susceptibility to HLA-A2 restricted MART1 specific CD8^+^ T cells was evaluated by the CD107a degranulation assay [61,62]. Briefly, target cells were co-incubated with effector cells at a 1:1 ratio at 37 °C. After one hour of incubation, an anti-CD107a Ab (Biolegend, San Diego, CA, USA) was added and after an additional 3 h, the effector cells were stained with the anti-CD3 and anti-CD8Ab (Biolegend) and analyzed on a Navios flow cytometer (Beckman Coulter, Brea, CA, USA). Effector cells alone were used to determine the spontaneous degranulation that was removed from the specific one.

Incubation with peptide-pulsed versus unpulsed T2 cells was used as a positive control for the functionality of the clone.

### 2.11. Immunohistochemical Staining of the Paraffin-Embedded Tissue Sections of Melanoma Patients

Formalin-fixed paraffin-embedded (FFPE) tumor samples were processed and analyzed at collaborating institutions in Zurich and Salzburg. The studies were performed according to the declaration of Helsinki and approved by the ethical committees of the University Hospital in Zurich (KEK-ZH-No. 647 and 800) as well as of the University Hospital in Salzburg (E-No. 2142). Four µm thick sections were cut from each FFPE tissue block and stained using the Dako Autostainer Plus platform (Agilent Technologies Inc., Santa Clara, CA, USA) as recently described [53].

Immunohistochemical stains were independently evaluated by three independent dermatopathologists. A consensus-based score was derived for every single evaluation. Cell counts were estimated by averaging at least ten high-powered fields (HPF, 400× magnification) representative of the entire tumor. The expression of TAP1 in tumor cells was graded into four categories regarding their frequency (0%, 1–10%, 11–30%, >30%). For the present study, TAP1^high^ (>30%) and TAP1^low^ (0–10%) lesions were processed as published by Lazaridou et al. (Oncoimmunol.2020 9,1:1–14) [56].

### 2.12. Next-Generation Sequencing Analysis

As previously described in the miTRAP method [35,36], miR can be eluted from the beads for downstream analyses, such as small RNA sequencing. Small RNA sequencing and data analyses were provided for two biological replicates of the miTRAP eluates of TAP1 3′UTR, together with the background and input controls by Novogene Co., Ltd. (Hong Kong, China).

The reads were mapped with bowtie1 [63] against human rRNA and tRNA databases. MiRs were detected and quantified with mirdeep * [64] from mirBase (v21) [65] and were represented as counts per miR loci taking into account that an miR can be encoded by multiple loci.

### 2.13. Functional and Pathway Enrichment Analyses

Gene Ontology (GO) analysis was performed using the respective database (http://www.geneontology.org/) enrichment analysis. Predicted target gene candidates of miRs identified in this study [66] were categorized regarding their function into the biological process (BP), molecular function (MF), and cellular component (CC) [67]. It provides all GO terms significantly enriched in the predicted target gene candidates of the enriched known as well as novel identified miRs compared to the reference gene background (in this study the MS2 control) as well as the genes corresponding to certain biological functions. The corrected *p*-value (*q*-value) of <0.05 and the gene count of ≥2 was chosen as a significant threshold [67]. 

### 2.14. Bioinformatics—Survival Analysis

In silico analysis was performed using the starbase v2.0 web tool (http://starbase.sysu.edu.cn/) [68,69] in order to predict the association of the expression of TAP1 and HLA class I molecules with the survival of melanoma patients. The statistical differences in the gene expression values between the patients’ groups with “high” and “low” mRNA expression levels were evaluated by ANOVA tests implemented in the web tool. The *p*-values were corrected for multiple testing according to the false discovery rate. All cut-off expression levels and their resulting groups were correlated with the patients’ survival and used for the generation of the Kaplan–Meier curves, which allowed to discriminate patients into “good” and “bad” prognosis cohorts. Kaplan–Meier analysis was performed to estimate the disease specific survival probability and distant metastasis free survival probability according to mRNA expression status using this dataset.

For this purpose, the “TCGA Skin Cutaneous Melanoma (SKCM)” dataset [70,71,72,73,74,75,76] was chosen and 440 unique melanoma patients were included for the analysis. For determination of high and low expression levels of TAP1 and HLA class I, the cut-off modus “median” divided the patients into two groups containing the same number of patients. The raw *p*-value significance was calculated for every graph with the web database and Pearson’s correlation with the transform 2log setting. The 2log expression ratio was compared and a linear regression was calculated. The expression pattern of TAP1 and HLA class I molecules were correlated to the clinical parameters. A *p*-value < 0.05 was considered as significant.

### 2.15. Statistical Analysis

Microsoft Excel 2010 (Microsoft Corporation, Redmond, WA, USA), SPSS version 15.0 and GraphPad Prism version 8 were used for analysis. A *p*-value < 0.05 was considered statistically significant using paired or unpaired *t*-tests respectively.

## 3. Results

### 3.1. Clinical Relevance of TAP1 and HLA Class I Molecules Regarding Survival of Tumor Patients

**Figure 1:**
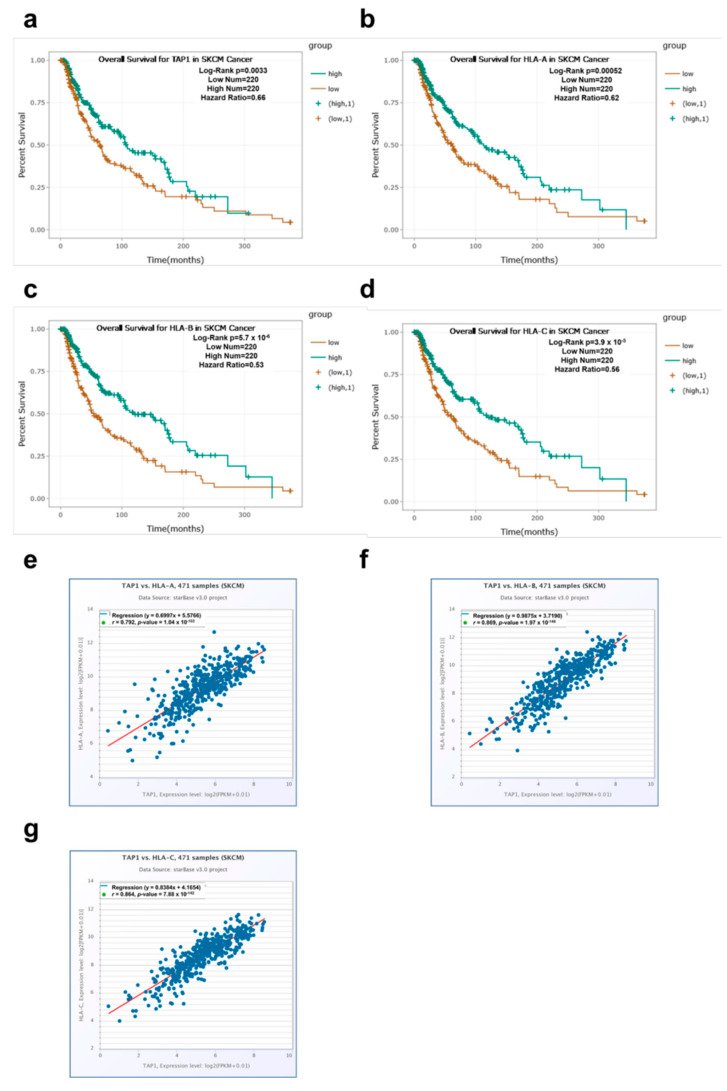
Correlation of TAP1 and HLA class I expression with the overall survival in melanoma patients. Kaplan–Meier estimation curves for overall survival (OS) probability of 440 individual melanoma patients based on the expression of TAP1 (**a**) HLA-A (**b**), HLA-B (**c**) and HLA-C (**d**) were generated by using the starbase v2.0 web tool and the “SKCM Cancer“ dataset. The raw *p*-values were based on log-rank tests and were calculated for every graph with the web database (http://starbase.sysu.edu.cn/). In addition, TAP1 expression was correlated with HLA-A (**e**), HLA-B (**f**) or HLA-C (**g**) mRNA expression in the melanoma patients using the same dataset.

As already shown by our group and others, a decreased expression of several HLA class I APM components, such as TAP1, HLA-A, HLA-B and HLA-C, has been associated with poor patient survival in several types of cancer, including cutaneous melanoma [2,77,78,79,80]. Using the “starbase” web tool (http://starbase.sysu.edu.cn/) and several available datasets including the “TCGA Skin Cutaneous Melanoma (SKCM)” dataset [70,81], the correlation between the expression of TAP1 and HLA class I loci was re-evaluated in order to determine the prognostic relevance of TAP1 (Table 1a), HLA-A (Table 1b), HLA-B (Table 1c) and HLA-C (Table 1d) expression patterns in different cancer types including skin cutaneous melanoma. These data demonstrated a correlation of high levels of TAP1 and HLA class I loci with an increased overall survival (OS) of tumor patients including melanoma, with the exception of cancers in immune privileged organs (brain, eye, thymus). As shown in Figure 1, higher TAP1 (a), HLA-A (b), HLA-B (c) and HLA-C (d) mRNA transcript levels correlated with an increased OS of melanoma patients. Furthermore, the expression of TAP1 and HLA-A (Figure 1e), TAP1 and HLA-B (Figure 1f) as well as TAP1 and HLA-C (Figure 1g) were associated.

### 3.2. Identification of New Candidate miR Targeting TAP1 Using the miTRAP Assay

**Figure 2:**
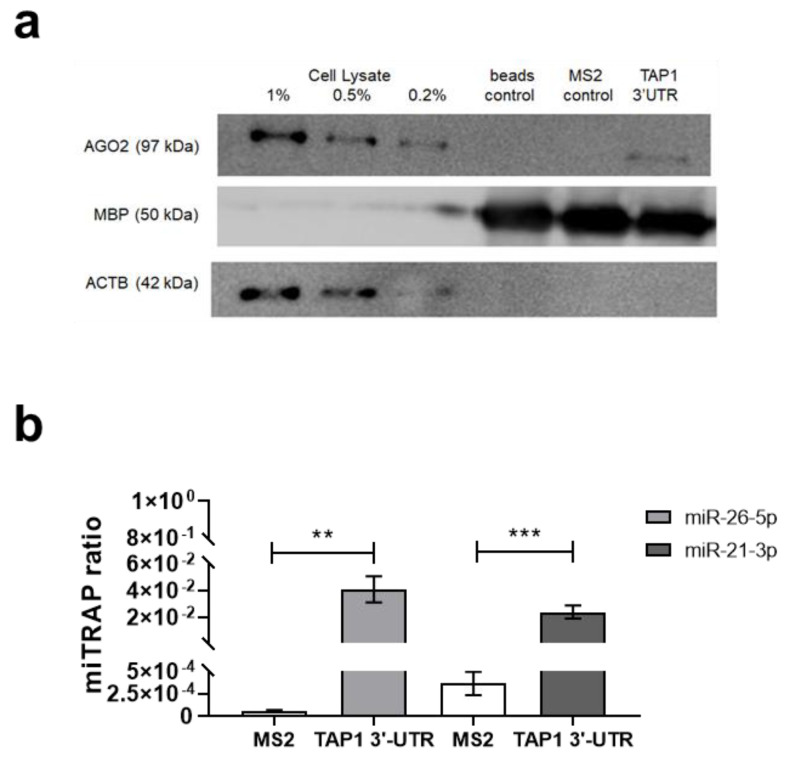
Detection of AGO2 at the 3′ UTR of TAP1 and presence of miRs in the miTRAP eluate. (**a**) Western blot-based detection of AGO2 was performed at the TAP1 3′ UTR in order to determine the presence of AGO2 in the cell lysate input control samples as well as on the target (TAP1 3′ UTR), control (MS2) RNA and in the amylose beads control (no RNA bait). Unspecific protein binding to RNA was excluded by the detection of ACTB, which lack in the beads control, MS2 control and target RNA. A signal for MBP of the MS2BP-MBP fusion protein is detected in the eluates, but not in the cell extract and served as control for equal loading of the MS2BP-MBP. (**b**) Enrichment of miRs in miTRAP eluates. Bar graph showing the miTRAP ratio of the two candidate miRs. The data were normalized to the input value, which was set to “1”. The bars show the enrichment of each miR on the MS2 in comparison to the TAP1 3′ UTR. For miR-26b-5p, a 700-fold enrichment compared to the MS2 control RNA (MS2) is detected, while for miR-21-3p, only a 50-fold enrichment is observed. Shown are the normalized mean ± SE from a minimum of three different biological replicates, ** *p* < 0.01 and *** *p* < 0.001 in unpaired *t*-test.

To identify miRs regulating TAP1 expression, the miTRAP method was employed [35,36,48,55]. Using a cell lysate of the melanoma cell line MZ-Mel2, the in vitro transcribed TAP1 3′ UTR, but not the MS2 control RNA nor the amylose resin beads loaded with MS2BP-MBP, specifically co-purified the RNA-induced silencing complex component argonaute 2 (AGO2) suggesting a putative posttranscriptional regulation of TAP1 expression by a binding of miRs (Figure 2a). The affinity purification of the bait RNA (TAP1 3′ UTR and MS2 control) was confirmed upon detection of the eluted TAP1 3′ UTR on a guanidinium thiocyanate denaturing agarose gel. The eluate-enriched miRs targeting the TAP1 3′ UTR from two biological replicates and the respective controls were subjected to small RNA sequencing.

The isolation of the miR fraction was based on size selection. Fractions of other RNA species, such as e.g., rRNA and tRNA, were excluded. The sequence length distribution demonstrated a major peak of 21–23 nt (Supplementary Figure S1). For analysis at the miR level, the transcripts per million (tpm) counts were used [36,53]. Selective binding of miRs to the TAP1 3′ UTR was determined relative to MS2 control by calculating the ratio of the respective tpm counts, indicating enrichment of the candidate miR as previously described [36,82]. In total, 352 of 2693 (13.07%) miRs determined by miRBase [65] were enriched in the TAP1 3′ UTR eluate when compared to the MS2 control (Supplementary Table S2). Among the enriched miRs, 2.27% (7 out of 352) were in silico predicted by 5 prediction tools, 3.98% (14/352) by 4 prediction tools, 3.98% (14/352) by 3 prediction tools, 13.64% (48/352) by 2 prediction tools, 49.72% (175/352) by 1 prediction tool and 26.42% (93/352) were not in silico predicted. These data suggest that the miTRAP allows a novel set of miR candidates to bind to the 3′ UTR. In order to select the candidate miRs, several criteria were applied, such as (i) that a specific binding site for the respective candidate miRs within the TAP1 3′-UTR should be predicted by at least four out of the six selected bioinformatic tools, (ii) the tpm counts observed in the TAP1 3′ UTR miTRAP eluate should be higher than 1000, while (iii) the tpm counts observed in the MS2 control miTRAP eluate should be less than 100 and (iv) the enrichment ratio should be higher than 50. Additionally, (v) a strong binding affinity of complementary structures between the putative miRs and the target TAP1 3′ UTR calculated as a high free binding energy and (vi) a high “miTRAP ratio”, defining the enrichment of miRs in the miTRAP eluate versus the input and determined by the ΔCt method were taken into consideration. 

In silico analyses were performed for 21 of the candidate miRs identified by miTRAP and RNA-seq using the available prediction tools miRWalk 2.0 [83,84], microrna.org [85], miRDB [86], TargetScan [87], RNA22 [88] and RNAhybrid [89]. These include miR-21-3p, miR-22-3p, miR-140-3p, miR-590-3p, miR-26b-5p, miR-532-5p and miR-26a-5p (Table 2). Despite their in silico prediction, the enrichment ratios of miR-1273f, miR-1301-3p, miR-140-3p, miR-151a-5p, miR-151b, miR-24-1-5p, miR-24-2-5p, miR-28-5p, miR-330-3p, miR-504-3p, miR-508-5p, miR-512-3p, miR-548b-3p, miR-597-3p and miR-708-5p were less than 50. Moreover, miR-26a-5p, miR-532-5p and miR-590-3p had lower binding energy and/or enrichment ratios than miR-26b-5p or miR-21-3p, while the miTRAP ratio of miR-22-3p was lower than the miTRAP ratios of miR-26b-5p or miR-21-3p. Therefore, based on the in silico prediction by the selected bioinformatics tools, the RNA sequencing results, the free binding energy, the miTRAP ratio and current literature, miR-26b-5p and miR-21-3p were selected as novel candidate miRs targeting the TAP1 3′ UTR for further analyses (Table 2). The enrichment of both candidate miRs was confirmed in the TAP1 3′ UTR eluates by RT-qPCR. Candidate miRs are presented as a miTRAP ratio of miR abundance in the target TAP1 3′ UTR or in the MS2 control eluates versus the input determined by the ΔCt method (Figure 2b).

Data from the GO analysis of the predicted target gene candidates from the enriched miRs of the miTRAP eluates are shown in Supplementary Figure S2, illustrating a categorization into biologic process, molecular function and cellular components. The molecular functions ATP binding and protein binding as well as the biologic process associated immune of genes located in the ER were highly enriched. Concerning the biologic processes, defense response and transmembrane/protein transport were also significantly enriched and are related to HLA class I APM components. The KEGG pathway analysis strengthens our assumption that miRs identified in the miTRAP eluate play a role in the regulation of antigen processing and presentation (Supplementary Table S3). 

### 3.3. Direct Interaction of miR-26b-5p and miR-21-3p with TAP1 3′ UTR

**Figure 3:**
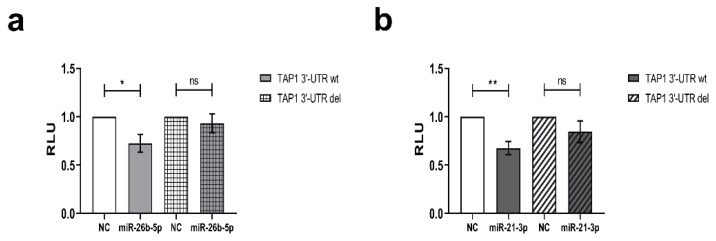
Identification of miR-26b-5p and miR-21-3p interaction with TAP1 3′ UTR. The dual luciferase reporter assay was performed with HEK293T cells using wt and del TAP1 3′ UTR as described in Materials and Methods together with miR-26b-5p (**a**) or miR-21-3p (**b**). Firefly luc (FFL) activities were internally normalized to Renilla luciferase activities yielding relative light units (RLU). Shown are the mean ± SE from 3 to 6 independent experiments upon normalization to the miR mimic NC. * *p* < 0.05, ** *p* < 0.01 in an unpaired *t*-test.

Using RNAhybrid [89], the binding affinity of complementary structures between the putative miRs and the target mRNA were calculated as a high free binding energy of −25.4 kcal/mol and −20.3 kcal/mol for miR-26b-5p or miR-21-3p and the TAP1 3′ UTR, respectively (Table 2 and Supplementary Figure S3a,b) indicating a high probability of interaction. Furthermore, the direct interaction between the two selected candidate miRs and the TAP1 3′ UTR was validated by the dual luc reporter assay. After transient transfection of HEK293T cells with the pMir-Glo vector containing the TAP1 3′ UTR in the presence of miR-26b-5p or miR-21-3p mimics, the luc activity significantly decreased upon overexpression of the miR in comparison to the miR mimic negative control (NC) (Figure 3a,b). As expected, the deletion of the binding sites of the candidate miR within the TAP1 3′ UTR altered neither the luc activity in the presence of the miR nor that of the NC (Figure 3a,b).

### 3.4. Downregulation of TAP1 Expression by miR-26b-5p and miR-21-3p

**Figure 4:**
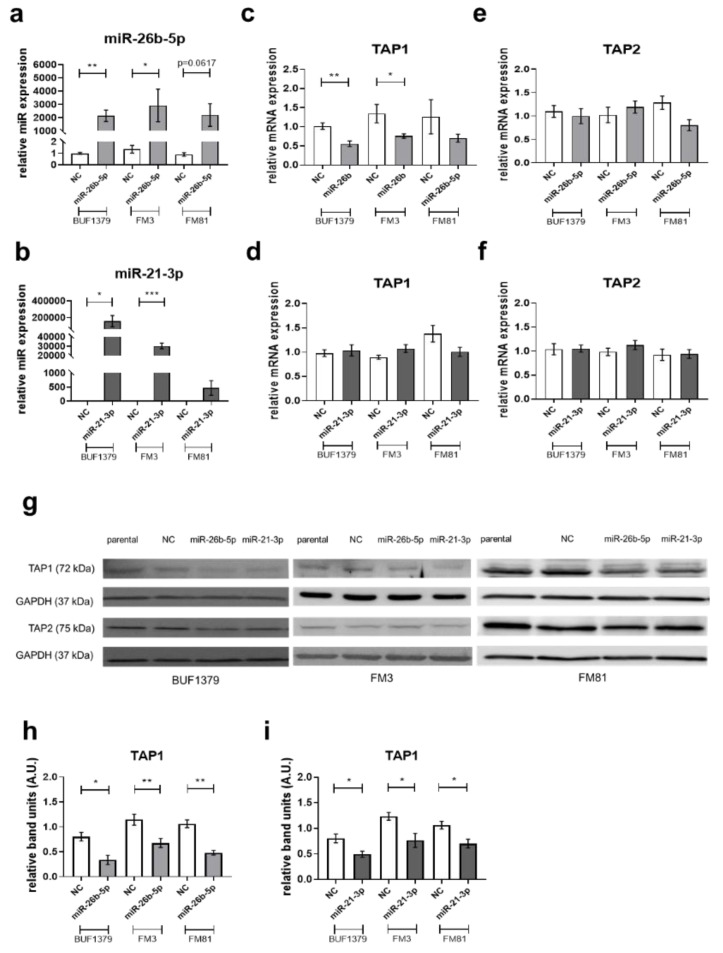
Effect of miRs overexpression on the expression of APM components in melanoma cell lines. BUF1379, FM3 and FM81 melanoma cells were left untreated (parental) or transiently transfected with miR mimics (miR-26b-5p or miR-21-3p) or miR mimic negative control (NC) as described in Materials and Methods. After 48 h of transfection, miR overexpression (**a**,**b**), as well as the mRNA expression levels of the indicated APM components, were determined by RT-qPCR (**c**–**f**). Protein expression was evaluated by Western blot (**g**–**i**). For quantification of Western blot results, the relative band density (A.U., arbitrary units) of transfectants was calculated to the respective parental melanoma cells and normalized to GAPDH expression. Shown are the normalized mean ± SE from a minimum of three different biological replicates and one representative Western blot, * *p* < 0.05, ** *p* < 0.01, *** *p* < 0.001 in an unpaired *t*-test.

**Figure 5:**
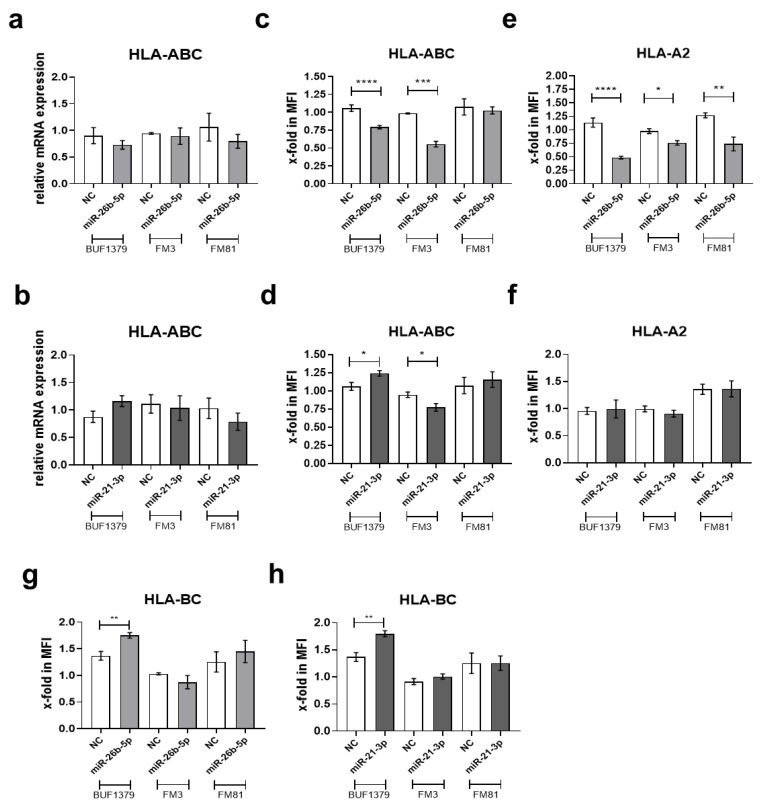
Effect of miRs overexpression on mRNA and surface expression of HLA class I in melanoma cell lines. BUF1379, FM3 and FM81 melanoma cells were transfected with 30 nM miR mimic negative control (NC) or miR mimics (miR-26b-5p or miR-21-3p) and after 48 h, the mRNA expression of HLA class I was determined by RT-qPCR (**a**,**b**) and the surface expression of HLA-ABC, HLA-A2 and HLA-BC was determined by flow cytometry (**c**–**h**). Shown are the normalized mean ± SE from a minimum of three different biological replicates, * *p* < 0.05, ** *p* < 0.01, *** *p* < 0.001 and **** *p* < 0.0001 in an unpaired *t*-test.

To determine whether miR-26b-5p and miR-21-3p, selectively co-purified with the TAP1 3′ UTR, regulate the TAP1 expression via binding to TAP1 3′ UTR, the respective miR mimics and NC were transiently transfected into the melanoma cell lines BUF1379, FM3 and FM81. Overexpression of both miRs was obtained in all three melanoma cell lines, but the level of overexpression varied among the three cell lines, based also on their constitutive miR expression level, with the highest levels for miR-21-3p in BUF1379 and FM3 cells. Cells transfected with the NC showed an miR expression pattern comparable to that of parental cells (Figure 4a,b). Overexpression of miR-26b-5p in BUF1379 and FM3 cells decreased the TAP1 mRNA and protein levels with approximately 30% when compared to controls (Figure 4c,g,h). In contrast, overexpression of miR-21-3p did not affect TAP1 mRNA levels (Figure 4d) in the melanoma cell lines analyzed, but interfered with TAP1 protein levels (Figure 4g,i) with a 25% decreased expression in melanoma transfectants compared to controls, potentially indicating a different type of action of miR-21-3p or miR-26-5p. As expected, the expression of other APM components, such as TAP2, was not affected by miR-26b-5p and miR-21-3p overexpression (Figure 4e–g). The miR-mediated downregulation of TAP1 was accompanied by decreased HLA class I surface antigen expression, but to a different extent among the melanoma cell lines. In contrast, HLA class I mRNA expression was not affected (Figure 5a,b) in the miR transfectants. As shown in Figure 5e, HLA-A2 surface expression was also significantly downregulated upon miR-26b-5p overexpression, whereas no effects were visible upon miR-21-3p overexpression. Interestingly, HLA-BC surface expression was upregulated upon overexpression of both miRs in BUF1379 cells (Figure 5g,h). Co-transfection of FM3 and FM81 cells with miR-26b-5p and miR-21-3p mimics was also performed and the expression levels of TAP1, TAP2 and HLA class I were evaluated by RT-qPCR, Western blot and/or flow cytometric analyses. Although both miRs were overexpressed during the co-transfection, no significant difference was observed compared to the single transfectants with miR-26b-5p or miR-21-3p, respectively.

### 3.5. Reversion of the miR Effect by Inhibition of miR-26b-5p and miR-21-3p

**Figure 6:**
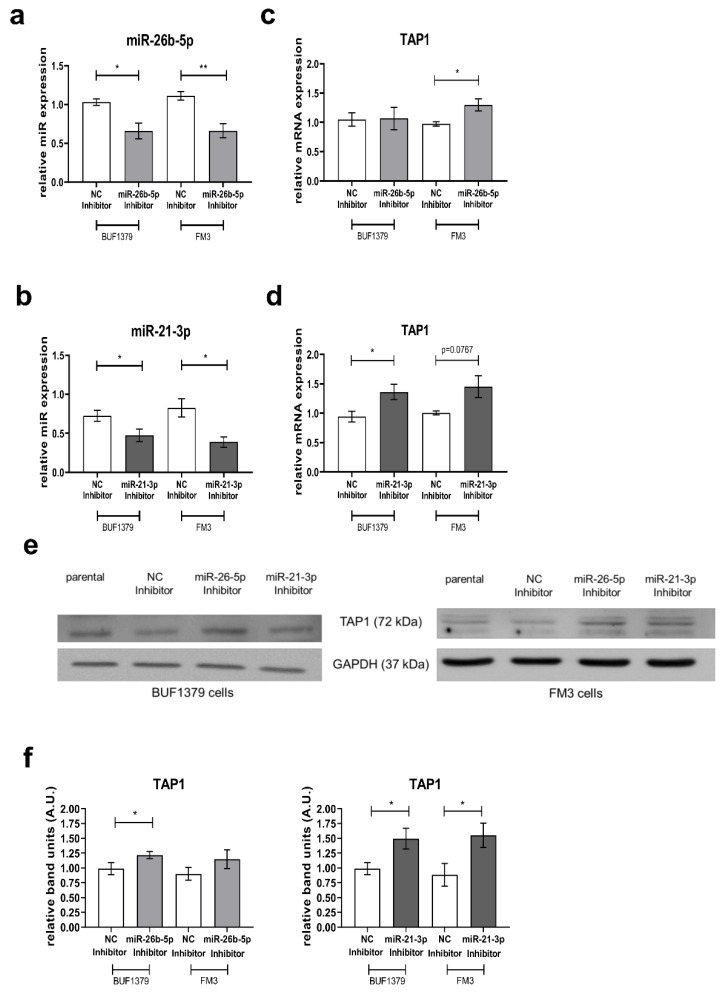

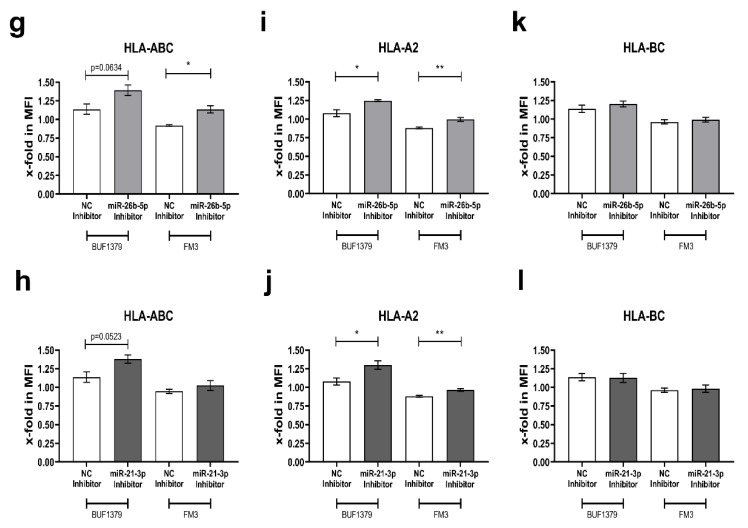
Effect of miRs inhibition on TAP1 and HLA class I expression in melanoma cell lines. BUF1379 and FM3 melanoma cells were left untreated (parental) or transiently transfected with the negative control inhibitor (NC Inhibitor) or the miR-26b-5p inhibitor or miR-21-3p inhibitor, respectively. After 48 h, miR inhibition (**a**,**b**) as well as TAP1 expression were determined on mRNA level by RT-qPCR (**c**,**d**). Protein expression was evaluated by Western blot (**e**,**f**) and flow cytometry (**g**–**l**). For quantification of Western blot results, the relative band density (A.U., arbitrary units) of transfectants was calculated to the respective parental melanoma cells and normalized to GAPDH expression. Shown are the normalized mean ± SE from a minimum of three different biological replicates and one representative Western blot, * *p* < 0.05, ** *p* < 0.01 in an un-paired *t*-test.

To evaluate the specific effect of the candidate miRs on TAP1 expression, miR inhibitors and an inhibitor control were transiently transfected into BUF1379 and FM3 cells. MiR expression levels were significantly reduced (between 30–50%) both in the BUF1379 and FM3 transfectants when compared to cells transfected with NC inhibitors or parental cells (Figure 6a,b). Thus, miR inhibitors efficiently down-regulated the endogenous miR expression in BUF1379 and FM3 cells. This was accompanied by increased levels of TAP1 protein (Figure 6e,f) and at least partially of TAP1 mRNA (Figure 6c,d). HLA class I surface expression was upregulated in both melanoma cell lines (Figure 6g,h). Inhibition of miR-26b-5p or miR-21-3p in BUF1379 and FM3 cells increased HLA-A2 surface antigens (Figure 6i,j), while HLA-BC surface expression remained unchanged (Figure 6k,l).

### 3.6. Correlation of the miR26b-5p-Mediated Downregulation of TAP1 with Decreased T Cell Recognition

**Figure 7:**
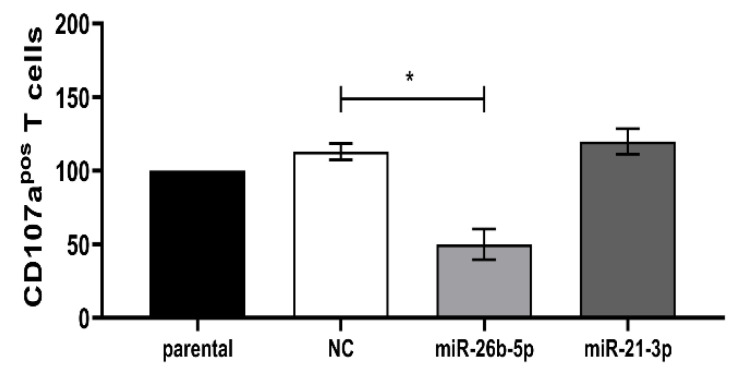
Effects of miR mimics on BUF1379 cell recognition by antigen-specific CD8^+^ T cells. MelanA/MART1-specific CD8^+^ T cells were incubated for 4 h with BUF1379 transfected with NC or miR mimics and evaluated for degranulation. Shown are the x-fold changes in CD107a positive cells from three independent experiments, * *p* < 0.05 in paired *t*-test.

To assess the functional relevance of the miR-induced suppression of TAP1 and consequently associated reduced HLA class I surface expression, the T cell-mediated recognition of miR transfectants was determined using a CD107a degranulation assay. Lower levels of CD107a positive T cells were found in response to BUF1379 cells overexpressing miR-26b-5p when compared to control transfectants (Figure 7).

### 3.7. Correlation between miR-26b-5p or miR-21-3p Expression with TAP1 and Immune Cell Infiltration in Melanoma Lesions

**Figure 8:**
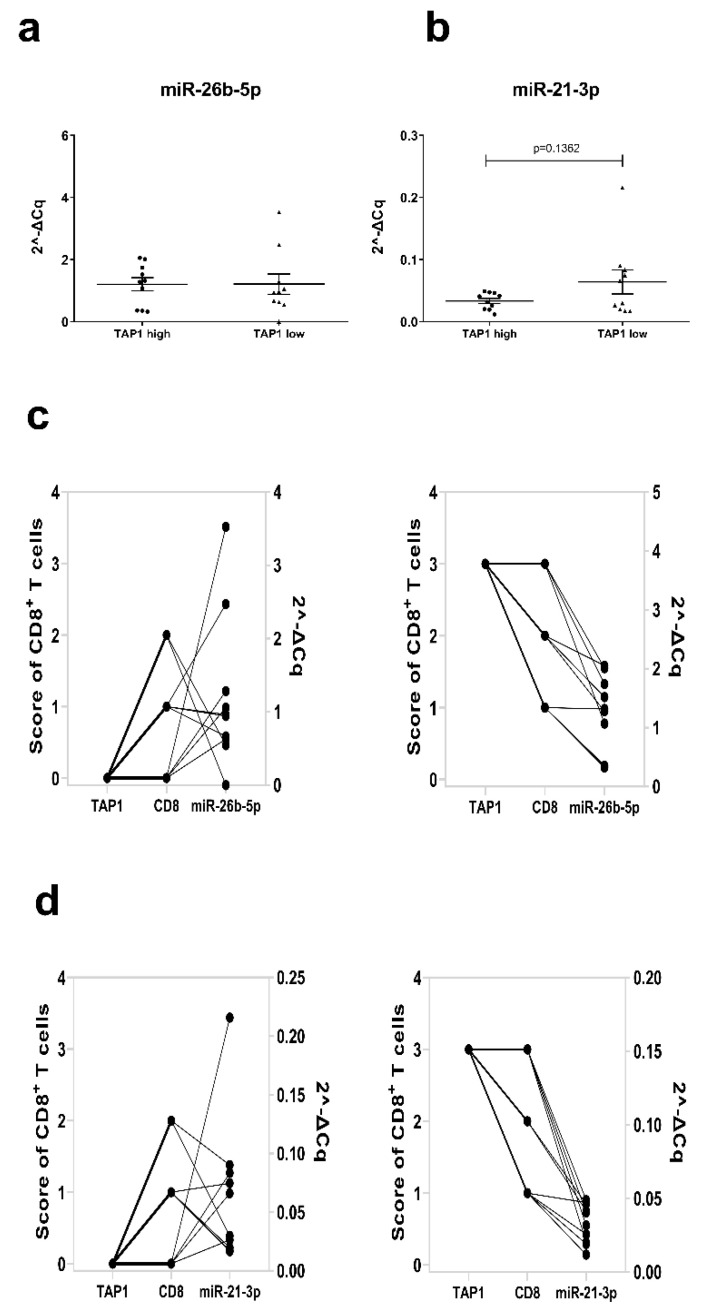
Inverse relation of miRs expression with TAP1 levels and CD8 infiltration in melanoma patients. Paraffin-embedded tissue sections from 20 primary melanoma patients were analyzed for miR-26b-5p or miR-21-3p expression levels by RT-qPCR and scored for TAP1 and CD8 expression as high or low by immunohistochemical staining. Comparison between TAP1 high (*n* = 10) and TAP1 low (*n* = 10) melanoma lesions is shown for miR-26b-5p (**a**) and miR-21-3p (**b**) expression. An overview of TAP1, CD8^+^ T cells (left *y*-axis) and miR-26b-5p expression (right *y*-axis) for each individual patient among the TAP1 high (left) and TAP1 low (right) group is provided (**c**). Respective graphs are provided for miR-21-3p (**d**).

To determine the in vivo translatability of our data, miR-26b-5p and miR-21-3p expression levels were evaluated by RT-qPCR analysis in 20 FFPE sections of human primary melanoma scored as TAP1 low (*n* = 10) or TAP1 high (*n* = 10) based on the immunohistochemical staining of the lesions with anti-TAP1 specific mAb. While no difference was detected for miR-26b-5p (Figure 8a), a negative trend was found between miR-21-3p and TAP1 expression in the melanoma specimens (Figure 8b), thereby supporting our in vitro data. Since the immune cell infiltration of the same melanoma samples was previously analyzed for the presence of CD8^+^ T cells by immunohistochemistry (IHC), it was possible to correlate TAP1 and miR expression to CD8 infiltration. As shown in Figure 8c,d, the TAP1 expression scores were directly correlated with the frequency of CD8^+^ immune cells, with TAP1^low^ and TAP1^high^ melanoma lesions exhibiting the low and high frequency of CD8^+^ T cells respectively. Furthermore, a direct link between TAP1^low^ and CD8^low^ infiltration with high miR-26b-5p and miR-21-3p expression and vice versa exists (Figure 8c,d).

## 4. Discussion

During the last few years, the role of HLA class I APM components has gained rekindled interest, since this pathway has been shown to be involved in the process of resistance to immunotherapies, including checkpoint inhibitors and adoptive T cell therapy [37,90,91,92]. High expression levels of major APM components including TAP1 and HLA class I loci were directly associated with a better outcome for most cancer patients, including skin cutaneous melanoma, with the exception of brain tumors, uveal melanoma and thymoma (Table 1). Furthermore, TAP1 expression was associated with HLA class I expression (Figure 1). A deficient/reduced expression of HLA class I components frequently occurred and was linked to either structural alterations or an impaired expression of components of the APM and IFN signal transduction, which are controlled at distinct levels [31,37,92,93,94,95]. 

In this context, a number of miRs have been shown to act as critical regulators of anti-tumor immune responses, in particular in the context of solid tumors. The miR-mediated processes include (i) the regulation of the recruitment and activation of immune cells in the TME, (ii) the expression of immune modulatory molecules and (iii) the secretion of immune suppressive or immune stimulatory factors by tumor and immune cells [96,97]. Thus, miRs might have the potential to enhance or inhibit specific immune cell populations or immune modulatory molecules in tumor cells, hence influencing the anti-tumor immune response and possibly improving the efficacy of immunotherapy in cancer patients.

During the last few years, some miRs have been reported to target APM components and HLA class I molecules suggesting an important role of posttranscriptional control in the antigen processing and presentation process [98]. miR-9, miR-125a, miR-148 and miR-27a have been identified to modulate directly or indirectly the MHC class I surface expression of tumor cells [21,23,99]. Since TAP1 is a key component of the HLA class I APM pathway and is important for proper HLA class I surface expression, this study aimed to identify miRs targeting TAP1 using an miR enrichment protocol in combination with small RNA sequencing [36,48] instead of miR arrays, which identified miR-200a-5p and miR-346 as targets of the 3′ UTR of TAP1 [28]. Interestingly, GO analysis of enriched miRs by miTRAP, in combination with RNA sequencing, identified respective target genes involved in ATP and protein binding and was located in the ER. This is in line with the features of TAP1, (i) which transport peptides ATP-dependently from the cytosol into the ER and (ii) is located in the ER membrane [100,101].

Among the identified miRs, miR-21-3p and miR-26-5p were selected for further analysis and were demonstrated to directly target the 3′ UTR of TAP1. The binding was confirmed by several in silico prediction tools and the dual luc reporter assay. Moreover, the overexpression of miR-21-3p or miR-26b-5p resulted in a reduced TAP1 protein expression in melanoma cells. As a consequence of the reduced transport of cytosolically processed peptides into the ER, which are required for the assembly of the stable trimeric MHC-I/peptide complex [102,103], melanoma cells overexpressing the respective mimics had a reduced HLA class I surface expression. Particularly, upon miR-26b-5p overexpression, HLA-A2 surface expression was specifically downregulated, resulting in a decreased recognition of the transfected melanoma cells by HLA-A2-restricted CD8^+^ T cells. Despite an overexpression of both miRs upon co-transfection in FM3 and FM81 cells with the respective miR mimics, the expression levels of TAP1 or the surface expression of HLA class I were not significantly different from that of the single transfectants with miR-26b-5p or miR-21-3p mimics respectively. Therefore, the results presented here provide an important mechanistic explanation for the reduced HLA class I expression on melanoma cells and its functional effect on immune cells.

The miR-21 family represents the prototype of oncomiRs and its expression is upregulated in various cancers, often correlating with tumor progression [104,105]. MiR-21-3p was among the top miRs isolated from invasive melanoma lesions and was differentially expressed between thin (0.75 mm) and thick (2.7 mm) melanoma, common melanocytic nevi and matched normal skin [106]. Its expression level correlated with the Breslow index, clinical stage and decreased survival of melanoma patients and increased from dysplastic nevi to melanoma and melanoma metastasis [107]. Since miR-21 has been shown to target cancer-relevant genes such as the phosphatase and tensin homolog (PTEN), programmed cell death protein 4 (PCDP4), reversion-inducing cysteine-rich protein with Kazal motifs (RECK) and signal transducer activator of transcription 3 (STAT3), it is involved in carcinogenic processes and might serve as a diagnostic and prognostic biomarker or as a therapeutic target for several cancer types [105]. In melanoma, miR-21 expression is upregulated when compared to nevi [108] and affects genes associated with proliferation (PTEN, PI3K, Sprouty, PDCD4, FOXO1, TIPE2, p53, cyclin D1), evasion from apoptosis (FOXO1, FBXO11, APAF1, TIMP3, TIPE2), genetic instability (MSH2, FBXO11, hTERT), increased oxidative stress (FOXO1), angiogenesis (PTEN, HIF1α, TIMP3), invasion and metastasis (APAF1, PTEN, PDCD4, TIMP3) [109]. This was partially confirmed by functional analysis. For example, miR-21 and its target PTEN demonstrated an inverse expression in melanoma lesions, while anti-miR-21 altered PTEN expression in melanoma cell lines. MiR-21-3p was strongly enriched in miTRAP eluates of TAP1 and proved to directly bind to the 3′ UTR, thereby decreasing TAP1 protein expression, which supported its reported oncogenic role in melanoma, similar to what has been described in other types of cancers [108]. 

MiR-26b-5p also plays an important role in different malignancies, but in contrast to miR-21-3p it has been suggested to have a tumor-suppressive activity [110]. It is downregulated in various tumors including head and neck squamous cell carcinoma, bladder, prostate and liver cancer [111,112,113,114,115] and was correlated to the process of epithelial-mesenchymal transition in hepatocellular carcinoma [113,116]. Ectopic expression of miR-26b-5p can inhibit proliferation, induce apoptosis, suppress angiogenesis and/or decrease tumorigenicity and is therefore involved in controlling carcinogenesis and tumor progression in hepatocellular and bladder cancer [113,117,118,119]. So far, little information exists on the function of miR-26b-5p in melanoma. In melanoma, miR-26b-5p expression is decreased in primary tumors when compared to benign nevi [106]. Recently, miR-26b-5p has been shown to target the MAPK and AKT/mTOR signaling pathways by binding to the 3′ UTR of TRAF5 or TRIM44, respectively [120,121], which are involved in the malignant progression of melanoma cells. However, no direct effects of miR-26b-5p on the expression of immune modulatory molecules have been described neither in melanoma nor in other tumor types. In this context, it is noteworthy that miR-26a-5p bind to the 3′ UTR of CREB, which is a known regulator of MHC class I [122]. MiR-26b-5p was one of the strongly enriched miRs we identified in miTRAP eluates of TAP1, verified by small RNA sequencing. By directly targeting the 3′ UTR of TAP1, miR-26b-5p decreased TAP1 mRNA and protein expression and negatively interferes with the immunogenicity of tumor cells demonstrating that miR-26b-5p could have, instead of the published tumor suppressive activity, also a tumor-promoting activity by including an immune escape phenotype in melanoma cells. This is in line with a recent report demonstrating that miR-26b is a negative regulator of the NF-ĸB pathway, which is important for the expression of many immune modulatory molecules including cytokines and APM components [123]. These data suggest a dual role for miR-26b-5p in tumors. 

## 5. Conclusions

Although further studies are required to provide more mechanistic insights into the link between miR-21-3p and miR-26b-5p high expression, HLA class I antigen presentation and CTL recognition, miR-21-3p/miR-26b-5p^high^ TAP1^low^ expression levels appear to be associated with a reduced CD8^+^ T cell infiltrate and a more aggressive behavior of primary melanomas. Thus, both miRs are multifaceted by affecting different hallmarks of cancer including the tumor cell-host immunologic interactions, thereby extending their critical role in tumorigenesis. This opens a novel avenue for the development of strategies to improve patients’ prognosis through enhancement of a response to (immuno)therapy and through avoidance of treatment resistance.

## Figures and Tables

**Table 1:** Correlation of the expression pattern of TAP1 and HLA class I loci in different cancer types with the patients’ survival.

a: TAP1					
Abbreviation	Type of Cancer	Number of Cases	Median Gene Expression	Correlation	*p*-Value
OV	ovarian serous adenocarcinoma	374	18.68	positive	0.00053
LGG	brain lower grade glioma	523	11.19	negative	0.0032
SKCM	skin cutaneous melanoma	440	41.39	positive	0.0033
UVM	uveal melanoma	80	9.02	negative	0.0069
COAD	colon adenocarcinoma	447	44.99	positive	0.044
THYM	thymoma	118	24.35	negative	0.1
UCEC	uterine corpus endometrial carcinoma	537	21.75	positive	0.56
THCA	thyroid carcinoma	509	12.61	negative	0.8
**b: HLA-A**					
LGG	brain lower grade glioma	523	133.35	negative	0.00012
SKCM	skin cutaneous melanoma	440	671.89	positive	0.00052
UCEC	uterine corpus endometrial carcinoma	537	523.84	positive	0.0074
UVM	uveal melanoma	80	502.23	negative	0.0081
THCA	thyroid carcinoma	509	468.03	positive	0.043
OV	ovarian serous adenocarcinoma	374	219.47	positive	0.059
THYM	thymoma	118	325.13	negative	0.063
COAD	colon adenocarcinoma	447	516.21	positive	0.26
**c: HLA-B**					
SKCM	skin cutaneous melanoma	440	620.14	positive	5.7 × 10^−6^
LGG	brain lower grade glioma	523	129.28	negative	0.00027
UVM	uveal melanoma	80	238.66	negative	0.0011
THYM	thymoma	118	390.69	negative	0.0026
OV	ovarian serous cystadenocarcinoma	374	300.77	positive	0.0069
UCEC	uterine corpus endometrial carcinoma	537	545.19	positive	0.053
COAD	colon adenocarcinoma	447	629.70	positive	0.18
THCA	thyroid carcinoma	509	503.74	positive	0.22
**d: HLA-C**					
LGG	brain lower grade glioma	523	117.10	negative	2.0 × 10^−7^
SKCM	skin cutaneous melanoma	440	458.38	positive	3.9 × 10^−5^
THYM	thymoma	118	260.27	negative	0.0081
UVM	uveal melanoma	80	247.30	negative	0.011
UCEC	uterine corpus endometrial carcinoma	537	406.42	positive	0.2
OV	ovarian serous cystadenocarcinoma	374	270.08	positive	0.4
THCA	thyroid carcinoma	509	334.74	positive	0.36
COAD	colon adenocarcinoma	447	461.01	positive	0.85

Shown are the correlation of TAP1 (a), HLA-A (b), HLA-B (c) and HLA-C (d) expression levels with the overall survival of patients with different types of cancer. The expression levels and overall survival data were obtained by pan-cancer analysis using the TCGA data sets at the starbase v2.0 web tool (http://starbase.sysu.edu.cn/).

**Table 2:** In silico prediction of selected, enriched miRs in the TAP1 3′ UTR miTRAP eluates.

miRBase Accession Number	miR	RNA-Seq Enrichment Ratio	miRWalk	Microrna.org	miRDB	TargetScan	RNA22	RNAhybrid	In Silico Prediction Tools	Binding Energy (kcal/mol)
MIMAT0020601	hsa-miR-1273f	0.9	yes	Yes	no	yes	no	yes	4	−30.1
MIMAT0005797	hsa-miR-1301-3p	9.2	yes	Yes	no	yes	yes	yes	5	−29.0
MIMAT0004597	hsa-miR-140-3p	1.3	yes	Yes	no	yes	yes	yes	5	−24.0
MIMAT0004697	hsa-miR-151a-5p	19.1	yes	Yes	no	yes	yes	yes	5	−26.1
MIMAT0010214	hsa-miR-151b	6.3	yes	Yes	no	yes	no	yes	4	−24.8
MIMAT0004494	hsa-miR-21-3p	1036.0	yes	Yes	yes	yes	no	yes	5	−20.3
MIMAT0000077	hsa-miR-22-3p	102.9	yes	Yes	no	yes	yes	yes	5	−21.6
MIMAT0000079	hsa-miR-24-1-5p	5.0	yes	Yes	no	yes	no	yes	4	−25.8
MIMAT0004497	hsa-miR-24-2-5p	17.2	yes	Yes	no	yes	no	yes	4	−25.9
MIMAT0000082	hsa-miR-26a-5p	74.1	yes	Yes	no	yes	no	yes	4	−25.1
MIMAT0000083	hsa-miR-26b-5p	92.1	yes	Yes	no	yes	no	yes	4	−25.4
MIMAT0000085	hsa-miR-28-5p	16.1	yes	Yes	no	yes	no	yes	4	−20.9
MIMAT0000751	hsa-miR-330-3p	21.7	yes	Yes	no	yes	no	yes	4	−24.8
MIMAT0026612	hsa-miR-504-3p	16.2	yes	Yes	no	yes	no	yes	4	−27.2
MIMAT0004778	hsa-miR-508-5p	3.4	yes	Yes	no	yes	no	yes	4	−25.5
MIMAT0002823	hsa-miR-512-3p	4.0	yes	Yes	no	yes	no	yes	4	−27.0
MIMAT0002888	hsa-miR-532-5p	65.5	yes	Yes	no	yes	no	yes	4	−20.5
MIMAT0003254	hsa-miR-548b-3p	1.3	yes	Yes	yes	yes	no	yes	5	−22.4
MIMAT0004801	hsa-miR-590-3p	289.2	yes	Yes	yes	yes	no	yes	5	−12.8
MIMAT0026619	hsa-miR-597-3p	2.7	yes	Yes	no	yes	no	yes	4	−23.5
MIMAT0004926	hsa-miR-708-5p	13.8	yes	Yes	no	yes	no	yes	4	−24.3

Six different prediction tools were used to predict binding of miRs to the 3′ UTR of TAP1. Sum demonstrates how many of the six tools used predicted the binding.

## Supplementary Materials

The following are available online at https://www.mdpi.com/2077-0383/9/9/2690/s1, Figure S1: Sequence length distribution of miTRAP eluates, Figure S2: The GO terms of candidate targets of miRs enriched in the TAP1 3′ UTR miTRAP eluates vs the MS2 control, Figure S3: RNAhybrid figures, Table S1: List of primers, Table S2: List of the enriched miRs in TAP1 3′ UTR miTRAP eluates, Table S3: Significantly affected processes, components or functions by the enriched miRs in mi-TRAP eluates of the TAP1 3′ UTR.

## Abbreviations

Ab: antibody; ACTB, β-actin; AGO2, argonaute 2; APM, antigen processing and presentation machinery; ATCC, American Tissue culture collection; BSA, bovine serum albumin; FCS, fetal calf serum; FFL, firefly luciferase; FFPE, formalin-fixed paraffin-embedded; GAPDH, glyceraldehyde-3-phosphate dehydrogenase; GO, gene ontology; HLA, human leukocyte antigen; HRP, horseradish peroxidase; HS, human serum; luc, luciferase; mAb, monoclonal antibody; MART, melanoma antigen recognized by T cells 1; MBP, maltose-binding protein; miR, microRNA; miTRAP, miRNA trapping by RNA in vitro affinity purification; NC, negative control; OS, overall survival; PBMC, peripheral blood mononuclear cell; RL, Renilla luc; RLU, relative light units; RTq-PCR, reverse transcribed quantitative PCR; TAA, tumor-associated antigen; TAP, transporter associated with antigen processing, tpn, tapasin; TCGA, The Cancer Genome Atlas; tpm, transcript per million; TME, tumor microenvironment; UTR, untranslated region.
