# Supplementary material for: Identification of microRNAs Targeting the Transporter Associated with Antigen Processing TAP1 in Melanoma
Source: J Clin Med. 2020 Aug 20;9(9):2690. doi: 10.3390/jcm9092690 (PMC7563967; doi:10.3390/jcm9092690)
Supplement: Supplementary file 1 [file jcm-09-02690-s001.pdf]

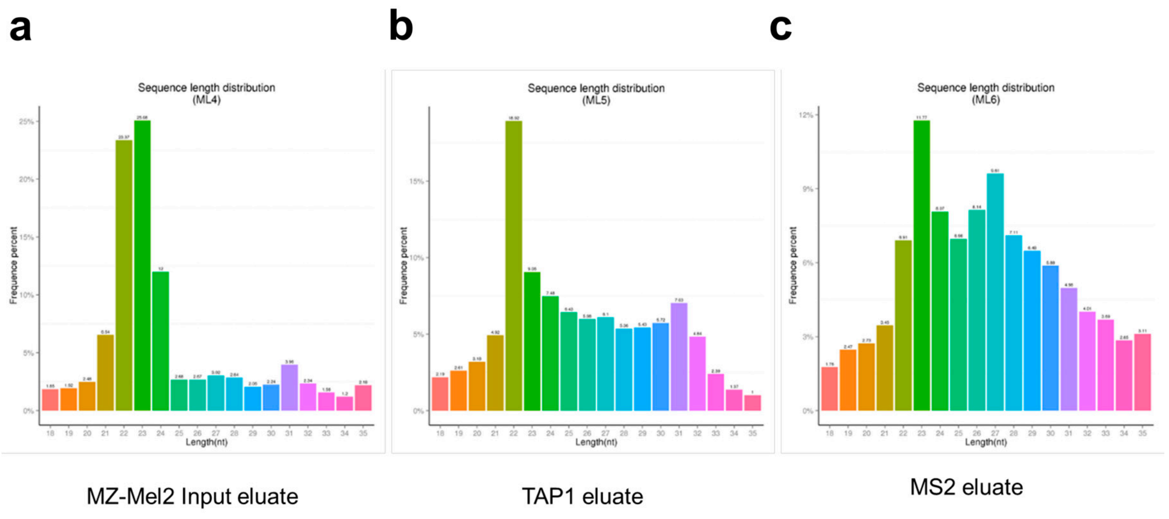

Figure S1. Sequence length distribution of miTRAP eluates.

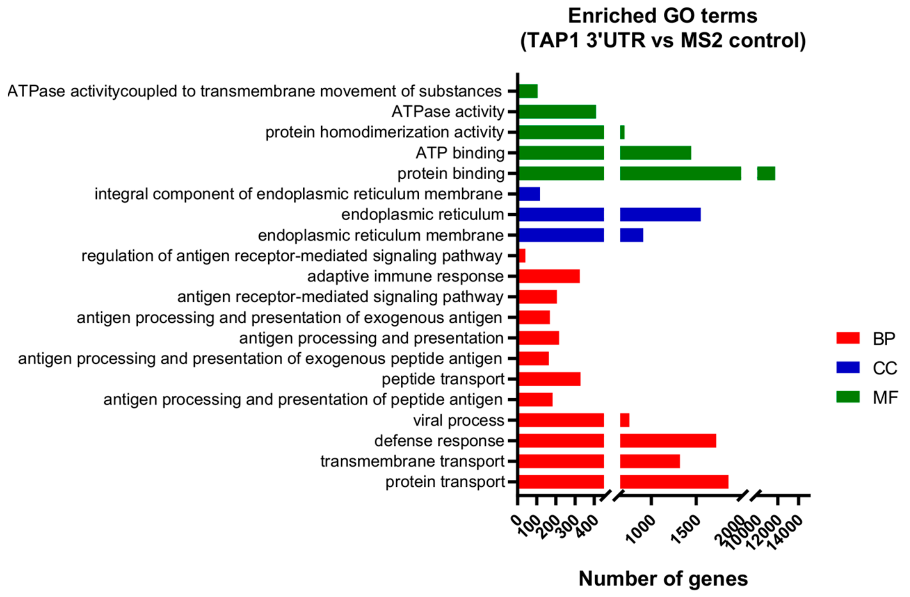

Figure S2. The GO terms of candidate targets of miRs enriched in the TAP1 3′ UTR miTRAP eluates vs the MS2 control.

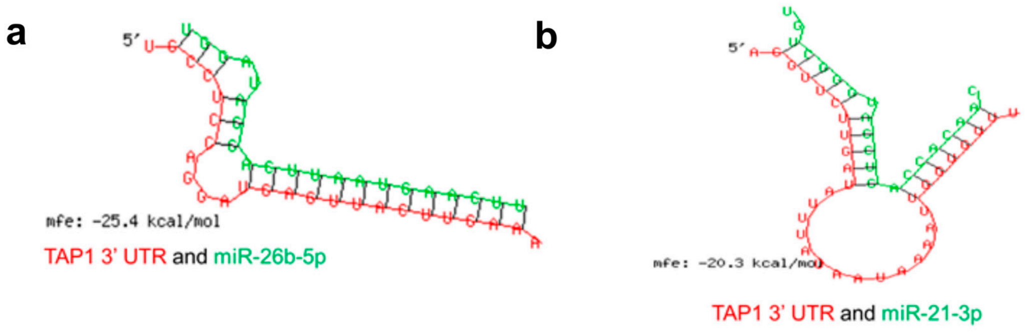

Figure S3. RNAhybrid figures.

Table S1. List of primers.

Name	Sequence
TAP1 2MS2 fwd	AAAGAATTCCTCCAGAATGAAAGCCTT CTC
TAP1 2MS2 rev	AAACTCGAGACAAAACACCAATTTTATTA
TAP1 3′UTR LUC fwd	AAAGCTAGCCTCCAGAATGAAAGCCTTCTC
TAP1 3′UTR LUC rev	AAAGTCGACACAAAACACCAATTTTATTA
del TAP1 3′UTR miR-26b-5p fwd	TTTGCCTTGAGTGTGTTACCTC
del TAP1 3′UTR miR-26b-5p rev	GCTGCCTACTCTGCAGCT
del TAP1 3′UTR miR-21-3p fwd	GTGTCGACCTGCAGGCAT
del TAP1 3′UTR miR-21-3p rev	ACAGGGTGTTTATGGGCC
SLRT miR-26b-5p	GTCGTATCCAGTGCAGGGTCCGAGGTATTCGCACTGGATACGACACCTAT
miR-26b-5p fwd	GCCCGCTTCAAGTAATTCAGG
SLRT miR-21-3p	GTCGTATCCAGTGCAGGGTCCGAGGTATTCGCACTGGATACGACACAGCC
miR-21-3p fwd	GCCCAACACCAGTCGATGG
general miR reverse primer	GTGCAGGGTCCGAGGT
RNU6A qPCR fwd	CGGCAGCACATATACTAAAATTGGA
RNU6A qPCR rev	AATATGGAACGCTTCACGAATTTGC
TAP1 qPCR fwd	GGAATCTCTGGCAAAGTCCA
TAP1 qPCR rev	TGGGTGAACTGCATCTGGTA
TAP2 qPCR fwd	CCAAGACGTCTCCTTTGCAT
TAP2 qPCR rev	TTCATCCAGCAGCACCTGTC
HLA-ABC fwd	GCCTACCACGGCAAGGATTAC
HLA-ABC rev	GGTGGCCTCATGGTCAGAGA
GAPDH fwd	GAGAAGGCTGGGGCTCATTTG
GAPDH rev	GGACTGTGGTCATGAGTCCTTC
ACTB fwd	TCCTGTGGCATCCACGAAACT
ACTB rev	GAAGCATTTGCGGTGGACGAT

Table S2. List of the enriched miRs in TAP1 3′ UTR miTRAP eluates.

miR	TAP1 3'UTR Biol. Repl. 1 (tpm)	MS2 control Biol. Repl. 1 (tpm)	TAP1 3'UTR Biol. Repl. 2 (tpm)	MS2 control Biol. Repl. 2 (tpm)	Average enrichement ratio
hsa-let-7a-5p	33050.40	5232.11	43199.70	5495.93	7.09
hsa-let-7b-3p	2.40	0.32	3.77	0.86	5.97
hsa-let-7b-5p	1393.96	1908.77	1829.08	2883.87	0.68
hsa-let-7c-5p	243.54	45.76	609.15	75.94	6.67
hsa-let-7d-3p	120.97	27.01	135.31	153.60	2.68
hsa-let-7d-5p	906.07	315.59	2049.02	435.77	3.79
hsa-let-7e-5p	282.80	314.63	352.55	331.36	0.98
hsa-let-7f-1-3p	0.80	0.95	1.62	0.00	0.84
hsa-let-7f-2-3p	0.80	0.64	0.00	0.00	1.26
hsa-let-7f-5p	56416.01	2169.06	65537.27	1922.58	30.05
hsa-let-7g-5p	17767.35	403.94	27442.59	749.88	40.29
hsa-let-7i-3p	206.69	0.32	123.99	0.00	650.36
hsa-let-7i-5p	61665.78	2135.37	62125.48	4393.98	21.51
hsa-miR-100-5p	2479.48	4404.85	1073.83	8869.94	0.34
hsa-miR-101-3p	10793.55	225.96	19595.31	478.92	44.34
hsa-miR-103a-3p	1788.91	48.94	1465.74	169.13	22.61
hsa-miR-103b	1275.39	46.08	1122.35	164.82	17.24
hsa-miR-105-5p	7.21	15.89	7.01	56.95	0.29
hsa-miR-106a-5p	3.20	0.64	0.54	0.86	2.83
hsa-miR-106b-3p	54.48	122.36	72.77	428.01	0.31
hsa-miR-106b-5p	5.61	17.48	11.32	57.82	0.26
hsa-miR-107	32.04	1.59	39.89	10.36	12.01
hsa-miR-10a-3p	15.22	1.91	8.63	15.53	4.27
hsa-miR-10a-5p	704.19	363.89	270.08	911.24	1.12
hsa-miR-10b-5p	66.49	9.85	8.09	296.84	3.39
hsa-miR-1180-3p	365.31	10.17	489.48	40.56	23.99
hsa-miR-122-5p	11.22	2.22	52.29	94.06	2.80
hsa-miR-1246	7.21	0.95	21.56	13.81	4.56
hsa-miR-1248	5.61	4.13	1.08	31.07	0.70
hsa-miR-1254	0.80	3.18	1.62	9.49	0.21
hsa-miR-125a-3p	1.60	7.95	1.08	16.40	0.13
hsa-miR-125a-5p	273.18	32.73	159.03	132.03	4.77
hsa-miR-125b-1-3p	58.48	113.46	8.63	75.07	0.32
hsa-miR-125b-5p	222.71	89.94	111.05	188.12	1.53
hsa-miR-1262	1.60	1.91	3.23	11.22	0.56
hsa-miR-126-3p	137.79	56.57	40.97	77.66	1.48
hsa-miR-126-5p	7.21	1.27	2.16	0.00	5.67
hsa-miR-1268a	6.41	13.35	9.16	48.32	0.33
hsa-miR-1269a	0.80	0.64	0.00	0.00	1.26
hsa-miR-1269b	1.60	8.58	9.16	34.52	0.23
hsa-miR-1273f	1.08	0.80	1.08	0.80	1.35
hsa-miR-1273g-3p	3.20	3.18	4.31	4.31	1.00
hsa-miR-127-3p	0.80	1.59	0.00	0.00	0.50
hsa-miR-1283	72.90	31.15	26.41	25.89	1.68
hsa-miR-128-3p	198.68	29.24	212.39	133.75	4.19
hsa-miR-1287-5p	34.45	1264.57	28.57	2159.02	0.02
hsa-miR-1293	1.60	0.32	1.62	5.18	2.68
hsa-miR-129-5p	5.61	2.86	0.54	8.63	1.01
hsa-miR-1296-5p	1.60	11.76	0.00	19.85	0.07
hsa-miR-1299	31.24	0.32	51.75	2.59	59.15
hsa-miR-1301-3p	149.81	9.53	168.19	63.86	9.17
hsa-miR-1303	1.60	0.95	7.01	15.53	1.07
hsa-miR-1307-3p	40.06	110.60	81.40	563.49	0.25
hsa-miR-130a-3p	24.03	1.27	4.85	1.73	10.86

hsa-miR-130b-3p	19.23	0.64	17.79	0.86	25.43
hsa-miR-130b-5p	81.71	5.40	59.30	20.71	8.99
hsa-miR-1323	277.99	171.94	323.98	247.66	1.46
hsa-miR-132-5p	1.60	6.36	0.54	31.93	0.13
hsa-miR-1343-3p	0.80	0.95	2.70	0.86	1.98
hsa-miR-135b-3p	6.41	1.91	1.08	24.16	1.70
hsa-miR-135b-5p	390.15	8.58	636.11	28.48	33.90
hsa-miR-138-1-3p	0.80	0.95	0.00	2.59	0.42
hsa-miR-138-5p	8.01	6.99	7.55	32.79	0.69
hsa-miR-139-3p	6.41	0.32	5.93	0.00	20.17
hsa-miR-139-5p	20.83	3.81	1.08	7.77	2.80
hsa-miR-1-3p	116.96	74.37	29.11	60.40	1.03
hsa-miR-140-3p	682.56	506.59	494.87	374.51	1.33
hsa-miR-140-5p	83.32	721.11	23.72	975.96	0.07
hsa-miR-143-3p	1093.54	44.18	19.41	37.11	12.64
hsa-miR-143-5p	13.62	0.64	0.54	0.00	21.43
hsa-miR-145-3p	19.23	0.64	0.00	0.86	15.12
hsa-miR-145-5p	129.78	3.50	0.54	2.59	18.67
hsa-miR-146a-3p	4.81	12.08	2.16	21.57	0.25
hsa-miR-146a-5p	76427.30	6196.03	111664.14	16426.52	9.57
hsa-miR-146b-5p	268.38	12.08	234.50	64.72	12.92
hsa-miR-148a-3p	3261.38	150.96	81.40	1851.82	10.82
hsa-miR-148b-3p	1971.57	33.37	2948.73	113.04	42.58
hsa-miR-148b-5p	35.25	1.59	14.02	6.04	12.25
hsa-miR-149-5p	2.40	0.64	22.10	2.59	6.16
hsa-miR-151a-3p	178.65	215.79	177.36	801.65	0.52
hsa-miR-151a-5p	141.00	5.08	117.52	11.22	19.10
hsa-miR-151b	1.60	0.32	6.47	0.86	6.27
hsa-miR-152-3p	87.32	3.81	50.13	12.94	13.39
hsa-miR-155-5p	10.41	16.53	21.02	47.46	0.54
hsa-miR-15b-3p	2.40	1.59	4.31	12.94	0.92
hsa-miR-15b-5p	12.82	1.91	2.16	1.73	3.99
hsa-miR-16-1-3p	1.60	0.32	0.54	15.53	2.54
hsa-miR-16-2-3p	88.12	6.67	103.50	43.15	7.80
hsa-miR-16-5p	20.03	35.59	19.95	22.44	0.73
hsa-miR-17-3p	3.20	0.32	2.16	0.00	10.08
hsa-miR-17-5p	209.89	28.60	127.22	69.90	4.58
hsa-miR-181a-2-3p	20.83	40.68	11.86	93.20	0.32
hsa-miR-181a-3p	15.22	13.98	8.09	20.71	0.74
hsa-miR-181a-5p	786.70	38.46	1341.75	195.88	13.65
hsa-miR-181b-5p	1423.60	31.78	2030.15	227.81	26.85
hsa-miR-182-5p	8024.87	147.78	2142.82	186.39	32.90
hsa-miR-183-5p	20.03	32.73	12.94	100.96	0.37
hsa-miR-184	1.60	0.32	0.00	6.04	2.52
hsa-miR-185-3p	51.27	2.86	121.29	7.77	16.77
hsa-miR-185-5p	116.16	23.52	315.90	88.02	4.26
hsa-miR-186-5p	55.28	152.87	32.34	1029.46	0.20
hsa-miR-18a-3p	1.60	0.32	1.62	2.59	2.83
hsa-miR-191-5p	306.03	217.70	145.55	978.55	0.78
hsa-miR-192-5p	2.40	6.67	0.54	46.60	0.19
hsa-miR-193a-5p	17614.33	4903.50	10624.59	4230.89	3.05
hsa-miR-193b-3p	1.60	1.59	3.23	7.77	0.71
hsa-miR-193b-5p	16.02	9.53	9.16	32.79	0.98
hsa-miR-194-5p	0.80	0.95	3.77	11.22	0.59
hsa-miR-195-3p	5.61	0.95	4.85	6.90	3.29
hsa-miR-196a-5p	83.32	178.93	86.25	310.65	0.37
hsa-miR-197-3p	10.41	3.18	10.24	12.08	2.06
hsa-miR-199a-3p	74.50	6.99	35.04	13.81	6.60

hsa-miR-199a-5p	19.23	1.59	2.16	5.18	6.26
hsa-miR-19a-3p	4.81	9.85	3.23	20.71	0.32
hsa-miR-19b-3p	1.60	17.48	4.85	39.69	0.11
hsa-miR-200a-3p	527.14	11.44	1.62	2.59	23.35
hsa-miR-200b-3p	115.36	4.13	1.62	5.18	14.12
hsa-miR-203a-3p	9.61	14.62	12.94	26.75	0.57
hsa-miR-203b-5p	9.61	14.62	12.94	26.75	0.57
hsa-miR-204-3p	9.61	20.02	9.16	25.89	0.42
hsa-miR-204-5p	84.12	20.98	60.38	74.21	2.41
hsa-miR-206	3.20	1.59	0.54	3.45	1.09
hsa-miR-20a-5p	263.57	56.57	155.25	184.66	2.75
hsa-miR-210-3p	1169.64	43.86	452.82	150.15	14.84
hsa-miR-2110	2.40	5.40	0.54	9.49	0.25
hsa-miR-21-3p	9884.28	6.04	3003.72	6.90	1036.01
hsa-miR-21-5p	80047.59	8474.41	41019.15	24493.94	5.56
hsa-miR-218-5p	23.23	3.81	0.00	12.94	3.05
hsa-miR-219b-5p	0.80	1.27	0.54	0.00	0.63
hsa-miR-221-3p	459.85	505.32	184.36	893.98	0.56
hsa-miR-221-5p	1254.56	51.17	921.28	110.45	16.43
hsa-miR-222-3p	554.38	384.87	354.17	780.94	0.95
hsa-miR-222-5p	4.01	2.54	0.00	3.45	0.79
hsa-miR-22-3p	4805.95	34.96	8198.22	119.95	102.91
hsa-miR-22-5p	1.60	3.81	1.08	19.85	0.24
hsa-miR-23a-3p	39.26	80.72	31.81	79.39	0.44
hsa-miR-23b-3p	36.85	15.89	29.11	17.26	2.00
hsa-miR-23b-5p	3.20	7.63	3.23	28.48	0.27
hsa-miR-24-1-5p	8.01	0.95	4.31	2.59	5.03
hsa-miR-24-2-5p	180.25	6.04	84.63	18.98	17.15
hsa-miR-24-3p	1211.30	281.26	1658.73	557.45	3.64
hsa-miR-25-3p	489.49	234.54	341.77	700.69	1.29
hsa-miR-25-5p	34.45	20.02	49.59	83.70	1.16
hsa-miR-26a-5p	138317.40	1420.29	168232.85	3316.20	74.06
hsa-miR-26b-3p	0.80	2.54	2.16	0.86	1.41
hsa-miR-26b-5p	5482.10	45.45	5433.86	85.43	92.12
hsa-miR-27a-3p	200.28	161.45	177.36	911.24	0.72
hsa-miR-27a-5p	90.53	54.35	35.04	189.84	0.93
hsa-miR-27b-3p	8253.99	751.62	3533.62	734.34	7.90
hsa-miR-27b-5p	8.01	28.29	8.09	108.73	0.18
hsa-miR-28-3p	385.34	1254.08	384.90	1564.47	0.28
hsa-miR-28-5p	128.98	5.72	234.50	24.16	16.13
hsa-miR-29a-3p	1282.60	240.26	422.09	529.83	3.07
hsa-miR-29b-1-5p	11.22	3.18	6.47	11.22	2.05
hsa-miR-29b-3p	123.37	4.13	10.24	2.59	16.91
hsa-miR-29c-3p	1.60	0.32	1.08	2.59	2.73
hsa-miR-301b-3p	3.20	0.64	1.08	0.86	3.15
hsa-miR-3074-5p	1103.15	278.40	1547.68	540.19	3.41
hsa-miR-30a-3p	48.87	66.74	29.11	81.11	0.55
hsa-miR-30a-5p	859.61	1514.05	271.69	1852.69	0.36
hsa-miR-30b-3p	8.81	8.90	13.48	28.48	0.73
hsa-miR-30b-5p	36.85	23.84	14.02	11.22	1.40
hsa-miR-30c-1-3p	0.80	2.54	1.62	0.00	0.32
hsa-miR-30c-2-3p	17.62	11.76	29.65	44.01	1.09
hsa-miR-30c-5p	399.76	552.04	127.22	1044.99	0.42
hsa-miR-30d-3p	13.62	10.81	10.78	15.53	0.98
hsa-miR-30d-5p	1854.60	1890.65	913.19	6161.24	0.56
hsa-miR-30e-3p	63.29	25.74	38.81	90.61	1.44
hsa-miR-30e-5p	151.41	160.18	63.61	440.95	0.54
hsa-miR-3158-3p	28.04	0.64	38.27	0.86	44.23

hsa-miR-3158-5p	24.03	0.64	34.50	0.86	38.90
hsa-miR-3180-3p	0.80	3.18	0.54	111.32	0.13
hsa-miR-3184-3p	668.94	3388.49	866.83	9410.99	0.14
hsa-miR-3184-5p	107347.52	367393.92	100236.87	379634.09	0.28
hsa-miR-3189-3p	0.80	1.91	1.62	3.45	0.44
hsa-miR-3200-3p	1.60	0.95	0.00	0.00	1.68
hsa-miR-320a	4228.34	314.95	3681.33	974.23	8.60
hsa-miR-320b	213.90	36.87	369.27	149.28	4.14
hsa-miR-320c	72.10	16.21	133.69	47.46	3.63
hsa-miR-320d	12.02	5.08	14.02	8.63	1.99
hsa-miR-324-5p	0.80	0.64	0.00	2.59	0.63
hsa-miR-32-5p	10.41	3.18	3.23	9.49	1.81
hsa-miR-328-3p	32.04	0.95	9.16	2.59	18.58
hsa-miR-330-3p	140.20	3.50	46.90	14.67	21.65
hsa-miR-331-5p	0.80	1.27	0.00	0.00	0.63
hsa-miR-339-3p	24.03	105.83	10.24	120.81	0.16
hsa-miR-339-5p	14.42	0.64	7.55	21.57	11.52
hsa-miR-33b-3p	0.80	0.64	1.08	5.18	0.73
hsa-miR-340-3p	6.41	7.95	4.31	19.85	0.51
hsa-miR-340-5p	103459.66	26.38	94496.28	76.80	2576.29
hsa-miR-342-5p	58.48	5.08	40.43	13.81	7.21
hsa-miR-345-5p	4.01	0.95	0.54	13.81	2.12
hsa-miR-34a-5p	8.81	0.64	8.63	2.59	8.60
hsa-miR-34c-5p	378.13	12.39	7.01	2.59	16.61
hsa-miR-3529-3p	1071.10	73.73	8276.93	1391.89	10.24
hsa-miR-3591-3p	11.22	2.22	52.29	94.06	2.80
hsa-miR-3605-5p	0.80	1.27	0.54	3.45	0.39
hsa-miR-3607-3p	0.80	1.27	0.54	6.04	0.36
hsa-miR-3609	0.80	0.95	1.08	0.86	1.04
hsa-miR-361-3p	12.82	42.90	10.78	97.51	0.20
hsa-miR-3615	3.20	3.18	2.16	13.81	0.58
hsa-miR-361-5p	37.65	17.16	71.70	9.49	4.87
hsa-miR-362-5p	7.21	4.77	7.01	19.85	0.93
hsa-miR-3653-3p	0.80	0.64	0.00	0.00	1.26
hsa-miR-365a-3p	15.22	8.26	20.48	31.07	1.25
hsa-miR-365a-5p	4.01	2.86	3.23	18.12	0.79
hsa-miR-365b-5p	4.01	2.54	5.39	27.61	0.89
hsa-miR-3681-5p	6.41	50.21	0.54	3.45	0.14
hsa-miR-3687	5.61	6.99	0.54	3.45	0.48
hsa-miR-3688-3p	1.60	0.32	1.62	0.00	5.04
hsa-miR-3689a-5p	0.80	5.72	1.08	16.40	0.10
hsa-miR-3691-5p	4.01	34.96	6.47	88.02	0.09
hsa-miR-374a-3p	12.82	0.32	14.55	5.18	21.57
hsa-miR-374a-5p	679.35	2.54	395.14	11.22	151.21
hsa-miR-374b-3p	12.02	1.91	25.88	20.71	3.78
hsa-miR-374b-5p	586.42	6.99	424.25	12.08	59.50
hsa-miR-374c-3p	586.42	6.99	424.25	12.08	59.50
hsa-miR-378a-3p	5701.61	1079.28	6194.49	4860.82	3.28
hsa-miR-378a-5p	15.22	3.50	8.63	7.77	2.73
hsa-miR-378c	85.72	32.42	258.76	195.88	1.98
hsa-miR-378d	18.43	10.81	85.71	69.03	1.47
hsa-miR-423-3p	108658.96	368044.80	101686.98	381420.33	0.28
hsa-miR-423-5p	668.94	3388.49	866.83	9416.17	0.14
hsa-miR-424-3p	16.02	20.66	11.32	47.46	0.51
hsa-miR-425-5p	12.02	19.70	7.55	32.79	0.42
hsa-miR-4485-3p	2.40	29.24	1.62	25.89	0.07
hsa-miR-4488	5.61	143.01	2.70	68.17	0.04
hsa-miR-450a-5p	0.80	2.54	2.16	6.90	0.31

hsa-miR-450b-5p	2.40	2.86	2.70	1.73	1.20
hsa-miR-451a	18.43	18.43	52.29	100.10	0.76
hsa-miR-4521	5007.03	232.96	3942.24	598.00	14.04
hsa-miR-454-3p	48.87	2.22	35.58	3.45	16.14
hsa-miR-454-5p	4.01	1.91	1.62	5.18	1.21
hsa-miR-455-5p	1029.45	55.62	429.10	169.13	10.52
hsa-miR-4677-3p	50.47	0.95	43.13	1.73	38.96
hsa-miR-4683	8.81	1.59	7.01	6.04	3.35
hsa-miR-4731-5p	1.60	0.32	0.00	2.59	2.52
hsa-miR-4746-5p	3.20	0.64	10.78	4.31	3.77
hsa-miR-484	49.67	5.08	132.61	15.53	9.15
hsa-miR-486-3p	0.80	1.91	17.25	16.40	0.74
hsa-miR-486-5p	0.80	1.91	17.25	17.26	0.71
hsa-miR-498	278.79	1.59	218.86	0.00	175.45
hsa-miR-500a-3p	87.32	43.86	194.07	72.49	2.33
hsa-miR-501-3p	22.43	23.84	44.20	81.98	0.74
hsa-miR-501-5p	1.60	4.13	4.31	10.36	0.40
hsa-miR-502-3p	37.65	41.95	54.99	56.95	0.93
hsa-miR-503-5p	1.60	1.59	1.08	0.86	1.13
hsa-miR-504-3p	9.61	0.32	2.16	1.00	16.20
hsa-miR-504-5p	4.01	13.98	1.62	20.71	0.18
hsa-miR-505-3p	0.80	1.59	1.62	10.36	0.33
hsa-miR-505-5p	0.80	1.91	1.08	8.63	0.27
hsa-miR-506-3p	546.37	6.36	554.17	9.49	72.17
hsa-miR-508-5p	3.20	0.64	12.40	6.90	3.42
hsa-miR-509-3-5p	15.22	23.20	15.09	50.05	0.48
hsa-miR-509-3p	6.41	11.12	6.47	29.34	0.40
hsa-miR-509-5p	5.61	3.50	3.23	7.77	1.01
hsa-miR-5096	0.80	0.64	0.00	0.00	1.26
hsa-miR-512-3p	7096.36	1386.92	5714.71	1945.88	4.03
hsa-miR-514a-3p	22.43	53.39	14.02	84.57	0.29
hsa-miR-514a-5p	1.60	1.27	1.08	2.59	0.84
hsa-miR-515-5p	48.87	15.57	17.79	31.07	1.86
hsa-miR-516a-5p	2066.90	12.71	1513.72	63.86	93.15
hsa-miR-516b-5p	51675.75	329.25	38020.29	607.49	109.77
hsa-miR-517-5p	4.01	2.22	3.23	3.45	1.37
hsa-miR-517a-3p	8627.31	185132.19	3375.68	84471.95	0.04
hsa-miR-517c-3p	464.65	12946.33	198.38	7803.37	0.03
hsa-miR-518a-3p	1.60	25.42	1.08	23.30	0.05
hsa-miR-518b	24.83	15.57	11.86	37.11	0.96
hsa-miR-518c-3p	6.41	3.18	2.70	3.45	1.40
hsa-miR-518c-5p	2043.67	18.43	1265.20	25.89	79.87
hsa-miR-518e-3p	3.20	3.50	2.16	2.59	0.87
hsa-miR-518f-5p	16.82	17.80	5.39	14.67	0.66
hsa-miR-519a-3p	769.88	8.26	150.40	3.45	68.37
hsa-miR-519b-3p	286.00	2.86	109.97	4.31	62.74
hsa-miR-519c-3p	178.65	1.27	78.17	5.18	77.81
hsa-miR-519c-5p	170.64	49.90	124.53	64.72	2.67
hsa-miR-519d-3p	13.62	29.87	3.23	20.71	0.31
hsa-miR-519d-5p	11.22	2.86	5.39	0.86	5.08
hsa-miR-520a-3p	85.72	34.96	128.84	79.39	2.04
hsa-miR-520a-5p	8.81	2.54	5.93	12.08	1.98
hsa-miR-520b	22.43	1.91	8.09	0.86	10.57
hsa-miR-520d-3p	24.83	0.95	15.09	2.59	15.94
hsa-miR-520d-5p	3.20	3.18	0.54	1.73	0.66
hsa-miR-520e	23.23	1.59	7.01	0.86	11.37
hsa-miR-520f-3p	1969.97	53.71	755.78	67.31	23.95
hsa-miR-520g-3p	13.62	20.34	4.85	15.53	0.49

hsa-miR-520g-5p	24.03	6.99	22.10	18.98	2.30
hsa-miR-520h	2.40	2.54	1.62	0.00	0.95
hsa-miR-522-3p	36.85	57.52	9.70	37.97	0.45
hsa-miR-523-3p	22.43	5.08	5.93	0.86	5.64
hsa-miR-524-5p	12.82	1.59	3.77	0.00	8.07
hsa-miR-525-5p	10.41	3.50	4.31	6.04	1.85
hsa-miR-526a	12.02	13.67	8.09	17.26	0.67
hsa-miR-526b-3p	14.42	0.64	2.16	0.00	22.69
hsa-miR-526b-5p	282.80	41.95	143.39	62.13	4.52
hsa-miR-532-3p	4.01	6.99	2.70	12.08	0.40
hsa-miR-532-5p	6358.53	60.70	5807.43	220.91	65.52
hsa-miR-542-3p	1.60	1.27	0.00	1.73	0.63
hsa-miR-548ay-3p	2.40	0.32	0.00	0.00	7.56
hsa-miR-548b-3p	2.07	1.00	0.59	1.00	1.33
hsa-miR-548o-3p	8.01	2.86	11.32	12.08	1.87
hsa-miR-550a-3-5p	0.80	1.27	5.93	2.59	1.46
hsa-miR-550a-5p	0.80	1.27	5.93	2.59	1.46
hsa-miR-551b-5p	4.81	3.18	10.24	20.71	1.00
hsa-miR-574-3p	38.45	18.12	35.58	20.71	1.92
hsa-miR-574-5p	576.81	3.18	348.24	4.31	131.10
hsa-miR-576-3p	1.60	3.18	1.08	12.08	0.30
hsa-miR-577	4.01	5.40	2.16	3.45	0.68
hsa-miR-584-5p	363.71	300.33	535.30	649.78	1.02
hsa-miR-589-5p	22.43	5.08	65.77	20.71	3.79
hsa-miR-590-3p	1176.85	3.18	718.05	3.45	289.16
hsa-miR-597-3p	2.40	0.64	4.31	2.59	2.72
hsa-miR-615-3p	142.60	23.52	368.19	121.67	4.54
hsa-miR-619-5p	4.01	2.86	2.70	7.77	0.87
hsa-miR-625-3p	1.60	3.81	3.77	18.98	0.31
hsa-miR-628-3p	1.60	0.32	1.62	0.86	3.46
hsa-miR-628-5p	0.80	4.45	1.62	6.90	0.21
hsa-miR-629-3p	0.80	1.91	0.54	5.18	0.26
hsa-miR-629-5p	230.72	22.25	459.83	80.25	8.05
hsa-miR-6505-5p	0.80	0.64	0.00	0.86	0.63
hsa-miR-6515-5p	18.43	0.64	5.39	0.00	28.99
hsa-miR-6516-3p	3.20	0.64	1.08	0.00	5.04
hsa-miR-6516-5p	0.80	1.59	0.00	0.86	0.25
hsa-miR-659-5p	0.80	1.27	0.54	0.86	0.63
hsa-miR-660-5p	67.29	10.17	45.82	13.81	4.97
hsa-miR-671-3p	12.82	18.75	12.94	53.50	0.46
hsa-miR-671-5p	374.13	1.27	176.82	4.31	167.64
hsa-miR-708-5p	1005.41	46.08	719.66	122.53	13.85
hsa-miR-744-5p	598.44	97.25	439.88	88.88	5.55
hsa-miR-7-5p	1081.52	74.37	8309.81	1409.15	10.22
hsa-miR-760	1.60	1.59	18.87	4.31	2.69
hsa-miR-7641	8.01	1.27	1.62	19.85	3.19
hsa-miR-767-5p	20.03	5.08	19.95	15.53	2.61
hsa-miR-769-3p	4.81	0.32	2.16	0.00	15.12
hsa-miR-769-5p	472.66	6.04	580.58	35.38	47.34
hsa-miR-7704	8.81	39.73	7.01	41.42	0.20
hsa-miR-7706	3.20	0.95	1.08	15.53	1.72
hsa-miR-877-5p	20.03	5.72	49.06	44.87	2.30
hsa-miR-92a-1-5p	60.08	20.34	32.34	121.67	1.61
hsa-miR-92a-3p	397.36	411.56	452.82	1316.81	0.65
hsa-miR-92b-3p	4.81	0.64	1.62	6.90	3.90
hsa-miR-93-3p	0.80	0.64	1.08	11.22	0.68
hsa-miR-93-5p	235.53	77.55	188.68	307.20	1.83
hsa-miR-9-3p	15.22	12.39	9.70	14.67	0.94

hsa-miR-941	37.65	41.00	70.08	249.38	0.60
hsa-miR-95-3p	2.40	2.22	0.54	3.45	0.62
hsa-miR-9-5p	208.29	169.39	113.74	444.40	0.74
hsa-miR-96-5p	2.40	6.67	1.62	13.81	0.24
hsa-miR-98-5p	987.79	83.90	1554.69	94.92	14.08
hsa-miR-99a-5p	133.79	19.07	13.48	29.34	3.74
hsa-miR-99b-3p	11.22	26.06	12.40	132.89	0.26
hsa-miR-99b-5p	69.70	115.37	52.83	516.89	0.35

Table S3. Significantly affected processes, components or functions by the enriched miRs in mi-TRAP eluates of Table 1. 3′ UTR.

GO_accession	Description	Term type	*q*-Value
GO:0005515	protein binding	MF	1.10×10^{-19}
GO:0005524	ATP binding	MF	1.10×10^{-19}
GO:0015031	protein transport	BP	1.10×10^{-19}
GO:0005789	endoplasmic reticulum membrane	BCP	8.20×10^{-11}
GO:0055085	transmembrane transport	BCP	9.73×10^{-09}
GO:0042803	protein homodimerization activity	MF	4.40×10^{-08}
GO:0005783	endoplasmic reticulum	CC	1.87×10^{-07}
GO:0006952	defense response	BP	1.85×10^{-03}
GO:0016032	viral process	BP	3.08×10^{-02}
GO:0016887	ATPase activity	MF	1.50×10^{-01}
GO:0048002	antigen processing and presentation of peptide antigen	BP	1.46×10^{-02}
GO:0015833	peptide transport	BP	7.64×10^{-03}
GO:0002478	antigen processing and presentation of exogenous peptide antigen	BP	2.56×10^{-02}
GO:0019882	antigen processing and presentation	BP	5.47×10^{-02}
GO:0019884	antigen processing and presentation of exogenous antigen	BP	7.39×10^{-02}
GO:0050851	antigen receptor-mediated signaling pathway	BP	4.35×10^{-02}
GO:0002250	adaptive immune response	BP	1.56×10^{-01}
GO:0042626	ATPase activity coupled to transmembrane movement of substances	MF	1.31×10^{-01}
GO:0030176	integral component of endoplasmic reticulum membrane	CC	2.39×10^{-01}
GO:0050854	regulation of antigen receptor-mediated signaling pathway	BP	2.78×10^{-01}
